# Characterization of the Protein and Carbohydrate Related Quality Traits of a Large Set of Spelt Wheat Genotypes

**DOI:** 10.3390/foods11142061

**Published:** 2022-07-12

**Authors:** Viola Tóth, László Láng, Gyula Vida, Péter Mikó, Marianna Rakszegi

**Affiliations:** Agricultural Institute, Centre for Agricultural Research, Brunszvik u. 2, 2462 Martonvásár, Hungary; toth.viola@atk.hu (V.T.); langlmv@gmail.com (L.L.); vida.gyula@atk.hu (G.V.); rakszegi.mariann@atk.hu (M.R.)

**Keywords:** breeding, diversity, GxE, heritability, spelt, *Triticum aestivum* subsp. *spelta*

## Abstract

Spelt wheat (*Triticum aestivum* subsp. *spelta* L.) is an underexploited hexaploid wheat species that has become an increasingly fashionable raw material of bakery products in the last decades, partly because of its ability to grow under organic agricultural conditions and partly because of the growing number of people following the trend of having a healthy diet. However, due to its difficult threshing, most research on spelt seed is based on a very limited number of genotypes. Therefore, we determined the physical, compositional, and breadmaking quality traits of 90 spelt genotypes in order to highlight the variation of these properties and to identify possible genetic resources for spelt improvement. The thousand kernel weight of the spelt genotypes ranged between 23.2 and 49.7 g, the protein content between 12.1% and 22.2%, the gluten index between 0.7 and 98.8, the dough stability between 0.0 and 19.6 min, and the starch damage between 6.3 and 19.4 UCD value. The average values showed that spelt has higher protein and gluten contents but weaker dough strength and stability than common bread wheat. The starch pasting temperature was also higher in spelt, but the starch damage was lower, resulting in lower water absorption. Some genebank accessions (MVGB142, 145, 353, and 525) and internationally available cultivars (Bohemia, Bodensonne, Black-Bearded, and White-Beardless) were identified as good genetic resources for improving the breadmaking-quality traits of spelt.

## 1. Introduction

The earliest cultivated wheat (*Triticum*) species were einkorn (*T. monococcum* L.), emmer (*T. turgidum* subsp. *dicoccum* (Schrank ex Schübler) Thell.), and spelt (*T. aestivum* subsp. *spelta* (L.) Thell.). The allohexaploid early spelt (*T. aestivum* subsp. *spelta*, BBA^u^A^u^DD) was originated from a cross between *T. turgidum* L. subsp. *dicoccum* (BBA^u^A^u^) and *Aegilops tauschii* Coss. (DD), in which the origin of the A^u^ genome was *T. Urartu* Tumanian ex Gandilyan, while the origin of the B genome was most likely an already extinct species from the *Aegilops* section Sitopsis, whose closest living progeny is the diploid (S genome) *Ae. speltoides* Tausch [1,2,3]. All of these early cultivated species were hulled. Three main evolutionary processes were important in the domestication of *Triticum* species: the first was the mutation of the brittle rachis (Br) gene to lose ear fragmentation at maturity (brbr), and it first appeared 12,000 years ago (when *T. monococcum* was domesticated); second was the natural mutation of the glume gene (Tg), which made the wild emmer and spelt easier to thresh (tgtg), about 10 and 8 thousand years ago, respectively; and the third mutation occurred in the density of the spikelets (q gene) and resulted in a more productive spike shape (QQ). Both modern durum wheat and bread wheat are carrying this combination (brbrtgtgQQ) of alleles, while spelt has a different allele combination: brbrtgtgqq [4,5,6,7,8].

Spelt is divided into two large geographic groups, Europe and the Asia. It has already been widely used in some areas of Europe (Germany, Austria, and Switzerland) between the 12th and 19th centuries [9], but it was replaced in the last century because of its low yield and tough glumes, and also because it could not be adapted to modern agricultural practices. In recent decades, however, there has been a growing demand for spelt among consumers, bakers, and farmers. Spelt has become popular due to the nutritional benefits of the grain, such as the high protein content, the high lipid content, and its more preferable fatty acid profile. Furthermore, ancient wheats (e.g., einkorn, emmer, and spelt) also have different resistant starch, lutein, phytochemicals, antioxidants contents, and compositions than modern wheat varieties and offer several health benefits [10,11]. From an agronomic point of view, spelt is more resistant to diseases caused by various pathogenic fungi, has better early soil coverage in spring, and has better nutrient use efficiency than wheat, so it requires less pesticides, herbicides, and fertilization, and no seed treatment is necessary before sowing, due to the presence of husk [12,13,14]. This also contributes to the beneficial health effects of spelt. At the same time, spelt has lower carbohydrate (68%) and fiber content (12%) than common wheat (75% and 13.4%, respectively) [15,16,17]. The starch of spelt can contain from 2% up to 21% more amylose than hard red winter wheat; thus, the gelatinization temperature is also higher in spelt (87–93 °C) than in wheat (84.6 °C) [18].

Spelt not only has higher protein content than wheat, but it also has a different storage protein composition. The ratio of gliadin to glutenin (Gli/Glu) was found to be between 2.8 and 4.0 in spelt, while this value ranges between 1.5 and 3.1 in common wheat, referring to the higher dough extensibility and lower bread volume of spelt [17,19,20]. Contents of celiac-related epitopes showed that the consumption of spelt is not safe in patients with celiac disease [21]. However, diet trends are shifting towards high-quality regional products that are not produced in large quantities but offer novel, interesting products with new flavors.

The aim of this study was to determine the variation of the protein- and starch-related processing quality traits in a larger set of spelt genotypes and to identify possible genetic resources for breeding purposes.

## 2. Materials and Methods

### 2.1. Plant Materials

Ninety winter spelt genotypes were collected from European countries (Belgium, Czech Republic, Germany, Hungary, and Switzerland) and Australia (Supplementary Table S1) having diverse morphological traits in spike type (awn/awnless), color (white/red/gray/black), or form (*T. aestivum*-QQ allele or *T. spelta*-qq allele type) and plant height. The bread wheat varieties ‘Glenlea’ and ‘Ukrainka’ were used as wheat controls; ‘Glenlea’ is a Canadian high-breadmaking-quality wheat variety with strong dough caused by 1Bx7 high-molecular-weight (HMW) glutenin subunit overexpression, while ‘Ukrainka’ is a high-milling-quality variety from Ukraine. These are well-known and widely studied international wheat varieties that are suitable for being standard varieties and have been used by breeders in many crossing programs.

### 2.2. Field Conditions

The 90 genotypes were grown in small plots at the Agricultural Institute of the Centre for Agricultural Research in Martonvásár, Hungary (latitude: 47°3′ N; longitude: 18°8′ E; altitude: 115 m) for three years (2017, 2018, and 2019). The plots were 4 m × 1.2 m and contained 6 rows spaced 20 cm apart. The genotypes were sown in two replicates. The soil has a clayey chernozem texture and a pH of 7.25. The soil contains 2.8% *w*/*w* humus, 210 mg/kg P_2_O_5_ and 210 mg/kg K_2_O. The previous crop was facelia in two years (2016/2017, and 2018/2019), while it was oil radish in 2017/2018. The annual average N intake as NPK combined fertilizer was 120 kg/ha active ingredient (Supplementary Table S2). The plots were treated with herbicide (4 L/ha U-46 D-fluid SL, 500 g/L 2-methyl-4-chlorophenoxyacetic acid; 40 g/ha Granstar 50 SX, 50% tribenuron methyl) and insecticide (0.2 l/ha Karate Zeon 5CS, contains 50 g/L λ-cyhalothrin) twice a year. No fungicides were applied. The temperature and the volume of precipitation were typical for Hungary during the 2017/2018 season, but with a high number of warm days (≥25 °C) during the growing period (Supplementary Table S3). The cumulative precipitation was almost the half of the 30-years average in season 2016/2017, with a relatively high number of cold and hot days. The weather conditions showed the highest absolute minimum temperature in season 2017/2018 (Supplementary Table S3). At the end of the growing season, the physical, compositional, and breadmaking quality traits were determined in the grain and the flour by collecting, threshing (Wintersteiger LD350, Wintersteiger AB, Arnstadt, Germany), cleaning (Haldrup DC-20, Haldrup GmbH, Ilshofen, Germany), and grinding 2 kg of seeds/sample from one field replicate.

### 2.3. Analysis of the Physical Traits and Milling

The test weight and thousand kernel weight (TKW) of the grains were determined [22] by FOSS Tecator 1241 (FOSS, Hilleroed, Denmark) and Marvin System (MarviTech GmbH, Wittenburg, Germany) instruments, respectively, and then the samples were conditioned to a moisture content of 15.5% and milled by using a Chopin CD1 Laboratory Mill (CHOPIN Technologies, Ville-neuve-la-Garenne, France). Wholemeal samples were prepared on a Perten Laboratory Mill 3100 instrument.

### 2.4. Analysis of the Compositional Traits

The crude protein content of wholemeal was measured by an Elementar Rapid N III Analyzer (Elementar Analysensysteme GmbH, Langenselbold, Germany), according to ICC 167 (1995) [23]. The wet gluten content of the flour was determined by using a Perten Glutomatic 2200 [24] (ICC 137/1, 1995) (Perten, Hamburg, Germany). The spread of gluten provides information about the proteolytic activity of samples following the change in gluten ball diameter during 1 h at room temperature. The starch content of the grain was estimated by using the NIR method with FOSS Tecator 1241 (FOSS, Hilleroed, Denmark) instrument [25] (ICC 202, 1995). The amount of mixed linkage β-glucan was measured in wholemeal by enzymatic digestion and spectrophotometry (Evolution 60S, Thermo Fisher Scientific, Waltham, MA, USA), according to AACC 32–23.01 [26]. Measurements were carried out in 2 replicates.

### 2.5. Analysis of Dough Properties

The properties of the dough (water absorption, dough development time, dough stability and dough softening, and quality number) were determined by the Brabender Farinograph instrument (Brabender, Duisburg, North Rhine-Westphalia, Germany), according to ICC 115/1 (1995) [27], in one replicate analysis. The gluten index (GI) was determined as described by the ICC 155 (1995) method [28]. Zeleny sedimentation was measured by the ICC 116/1 method (1997) [29] with a SediCom System [30] in two replicates.

### 2.6. Starch Properties

Falling Number was measured by Perten Falling Number System 1500 (AACC 56–81B) [31]. Pasting viscosities were determined by a Rapid Visco Analyser (RVA-3D, Newport Scientific Pty. Ltd., Warriewood, NSW, Australia). A 20-min pasting profile was used, consisting of a 2-min hold at 50 °C, a 6-min heating period, a 4-min hold at 95 °C, a 4 min cooling period to 50 °C, and a 4-min hold at 50 °C [32,33]. Silver nitrate (12 mM) was used as an enzyme inhibitor. Starch damage was measured by the Chopin SDmatic instrument (Chopin Technologies, Villeneuve-la-Garenne, France) and determined according to the standard method of ICC 172 [34]. The damage rate of starch is given at 14% flour moisture and 12% protein content expressed in Chopin Units (UCD). Measurements were carried out in one replicate analysis.

### 2.7. Statistical Analysis

Least significant differences and correlations were calculated by using the Microsoft Excel 2013 software (Microsoft Corporation, Redmond, WA, USA). Construction of the principal component analysis (PCA) was carried out in Statistica 6.0 (TIBCO Software Inc., Palo Alto, Santa Clara, CA, USA). One-way ANOVA, Tukey’s post hoc test, hierarchical cluster analysis (with Ward’s Method and Euclidean distance), and Linear Mixed Model analysis (using the restricted likelihood algorithm, REML) were performed by using SPSS Statistics 27.0 software (SPSS Inc., Chicago, IL, USA). Linear Mixed Model analysis was based on Virk et al. [35]. A total of three years was considered as a different environment (E) for each genotype (G). In this model, replication was the random factor. The repeatability, genotypic variance, and variance of the GxE interaction were evaluated for each trait. Repeatability (broad-sense heritability) was calculated as the ratio of genotypic to phenotypic variance [36].

## 3. Results

### 3.1. Diversity of the Physical, Compositional, and Breadmaking Quality

The average test weight of spelt was 70.5 kg/100 L, while the thousand kernel weight was 36.9 g, with the ranges of 58.9–81.9 and 23.2–49.8, respectively (Table 1). The average width of the spelt kernels (2.9 mm) was lower, while its length was higher than those of the wheat controls (‘Glenlea’ and ‘Ukrainka’). The flour yield after milling was very variable in the case of spelt, in a range from 31.8% to 75.1% and having a 58.2% value in average. The average flour yield of spelt was similar to that of the wheat controls (Table 1).

The compositional properties of spelt could be characterized by a higher average protein (18.9%) and gluten (44.7%) content than those of wheat with a lower amount of starch (52.9%) but similar level of β-glucans (6.54 mg/g). At the same time, the variation of the amount of these components was high in spelt and ranged between 12.1% and 22.2% (protein), 29.3% and 59.8% (gluten), 49.2% and 57.8% (starch), and 4.5% and 8.4% (β-glucan).

Although the protein and gluten contents were high in spelt, the quality traits related to them showed low values. Therefore, the average gluten spread was 7.8 mm/h, which was 2.5 and 1.1 mm/h in the case of the wheat controls. The GI was 59.2, while being 97.3 and 99.7 in ‘Glenlea’ and ‘Ukrainka’, respectively. The average Zeleny sedimentation was 34.7 mL, with 48.0 mL (‘Glenlea’) and 45.6 mL (‘Ukrainka’) in wheat controls. The farinograph parameters also showed low average values in spelt compared to wheat; thus, the average dough development time was 4.7 min, the dough stability was 9.5 min, the softening of the dough was 76.0 FU (farinograph unit), the water absorption was 55.9%, and the quality number was 59.7 on average. The variation in these traits were high in spelt and changed between almost the technically possible ranges in the case of GI, dough-development time, dough stability, and quality number. Meanwhile, the gluten spread changed between 1.0 and 20.5 mm, the Zeleny sedimentation between 16.0 and 73.0 mL, the dough softening between 0.0 and 306.0 FU, and the water absorption between 51.3 and 63.3%.

Starch properties were characterized by the falling number, the viscosity parameters, and the starch damage. The average falling number was 383.2 s and ranged from 103 to 613 s (with 416 and 428 s for ‘Glenlea’ and ‘Ukrainka’, respectively). The average peak viscosity was 3706 cP, with 1751 cP trough- and 3824 cP final-viscosity values. The average breakdown and setback values were 1954 cP and 2073 cP, respectively. The average pasting temperature was higher in spelt (63.5 °C) than in the wheat controls (61.6 and 61.1 °C, respectively, for ‘Glenlea’ and ‘Ukrainka’). The average pasting time was 9.0 min. As the kernel hardness of spelt was rather soft, the average damage of starch (12.7) was found to be lower in spelt than in wheat (17.3 and 18.8 for ‘Glenlea’ and ‘Ukrainka’, respectively), but the variation of starch damage was high and changed between 6.3 and 19.4.

### 3.2. Variance and Heritability

**Figure 1:**
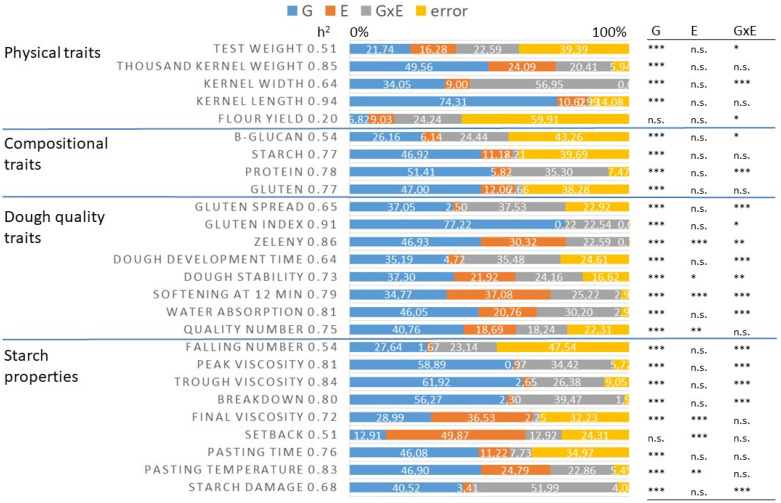
The relative contribution of genotype (G), environment (E-3 years), and genotype × environment interaction (G × E) for the total sum of squares to the physical, compositional, and breadmaking quality traits of spelt wheat and the broad-sense heritability of each traits (Martonvásár, Hungary, 2017–2019, *n* = 270); n.s. = non-significant; *, **, and *** significant at 0.5, 0.1, or 0.01 probability levels.

The effects of the spelt genotype (G) and the year as environment (E) and the interaction of these two factors (G × E) were examined for each trait. The broad-sense heritability values were also calculated (Figure 1).

The genotype determined 21.7% of the total variance in test weight, 49.5% in thousand kernel weight, 34.0% in kernel width, 74.3% in kernel length, and 6.8% in flour yield. The heritability values of these traits were 0.51, 0.85, 0.64, 0.94, and 0.2, respectively. The effect of the environment was relatively low for these traits, but a significant GxE effect could be observed on kernel width (56.9%) (Figure 1).

The genotype determined the protein, starch, and gluten contents at a similar extent, contributing to the total variance, respectively, in 51.4, 46.9, and 47.0%, and the heritability values of these traits were also similarly high (0.78, 0.77, and 0.77, respectively). A significant effect of G × E was found only for protein content. In the case of β-glucan content, the genotype effect was significant, but it was also relatively less determinant (26.1%), while its heritability value (0.54) was also low.

From the traits responsible for dough quality, the total variance of GI was the most determined by G (77.2), and the heritability of this parameter was also the highest (0.9). Furthermore, the variance of the farinograph parameters, the Zeleny sedimentation, and the gluten spread were determined by G in 34.7–46.9%, with 0.64–0.86 heritability values. The contribution of the environment to the total variance was significant in the case of dough softening and Zeleny sedimentation (18.6% and 37.0%, respectively), while significant GxE interactions were found in the case of gluten spread, dough development time, dough softening, and water absorption.

Starch properties were even more determined by G, especially the peak viscosity (58.8%), trough viscosity (61.9%), and the level of viscosity breakdown (56.2%), while their heritability was also high, at values of 0.81, 0.84, and 0.80, respectively. At the same time, the setback of viscosity (49.8%) and the final viscosity (36.5%) were more dependent on E, and their heritability values were the lowest (0.51 and 0.72, respectively). The GxE interaction significantly influenced the falling number, peak and trough viscosity, breakdown, and water absorption. The genotype determined 46% of the total variance in the case of pasting time and temperature, with 0.76 and 0.83 heritability values, respectively. The variance of starch damage was determined in 40.5% by G, while the effect of GxE was also significant on it (51.9%). The heritability of starch damage was 0.68.

### 3.3. Hierarchical Cluster Analysis

**Figure 2:**
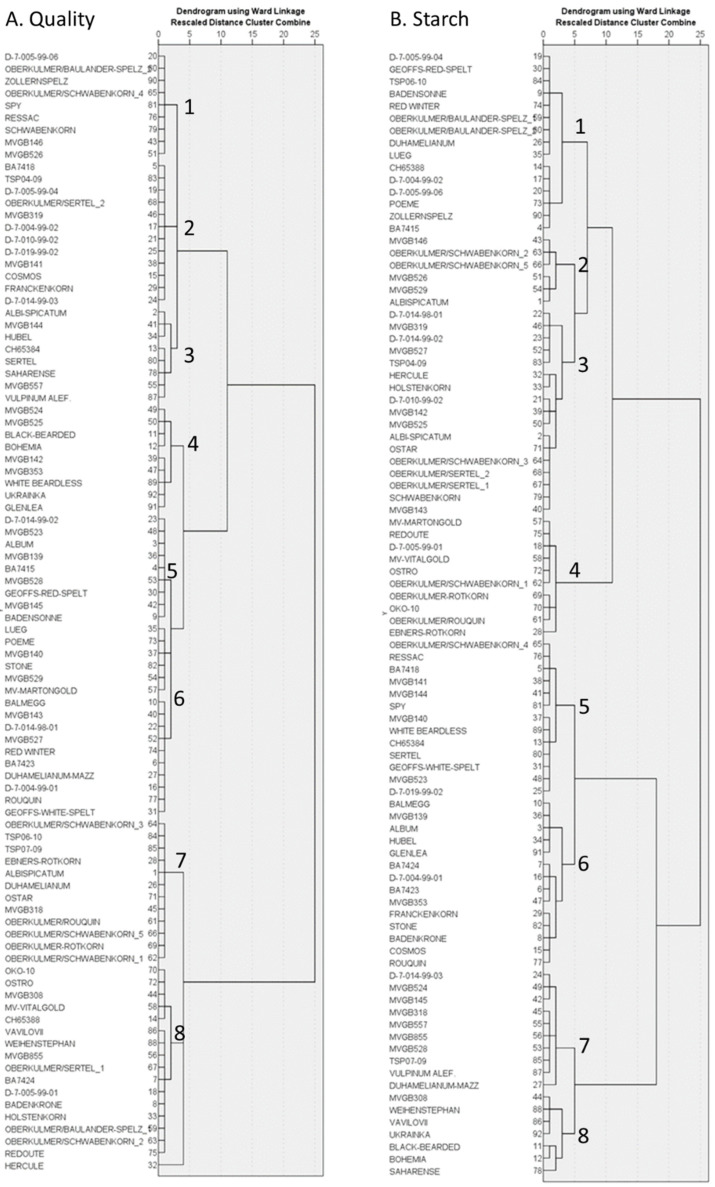
Hierarchical cluster analysis based on the quality traits (**A**) and the starch properties (**B**) of the flour of 90 spelt genotypes examined in Hungary (Martonvásár, 2017–2019).

**Figure 3:**
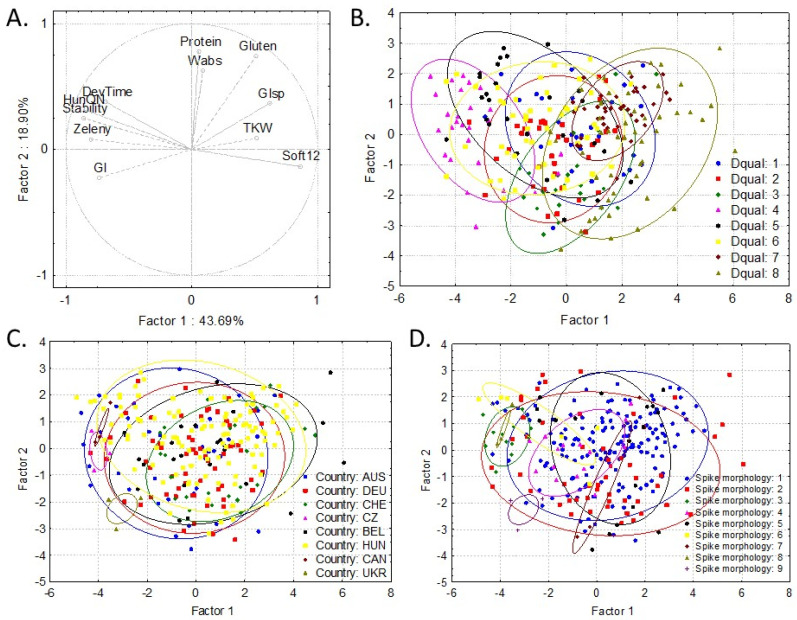
Principal component analysis based on the breadmaking quality traits and including protein and gluten content and TKW of spelt (**A**) and grouped by the groups formed by the hierarchical cluster analysis (**B**), country (**C**), and spike morphology (**D**) groups (where the most determinant traits contributed to Factor 1 was dough stability (−0.8591), dough softening (0.8607), farinograph quality number (−0.8674), and Zeleny sedimentation (−0.8019), while it was protein content (0.7798), gluten content (0.7416), and water absorption (0.6277) to Factor 2). DevTime, dough development time; GI, gluten index; Glsp, gluten spread; HunQN, farinograph quality number; Soft12, farinograph dough softening at 12 min; stability, farinograph dough stability; TKW, thousand kernel weight; Wabs, water absorption of the flour; Zeleny, Zeleny sedimentation; Dqual, quality groups on dendrogram (Figure 2a); AUS, Australia; BEL, Belgium; CAN, Canada; CHE, Switzerland; CZ, Czech Republic; DEU, Germany; HUN, Hungary; and UKR, Ukraine. Spike morphology: (1) awnless, red, spelt-type spike; (2) awnless, white, spelt-type spike; (3) awned, black, spelt-type spike; (4) awned, red, spelt-type spike, (5) awned, white, spelt-type spike; (6) awnless, gray, spelt-type spike; (7) awnless, gray, wheat-type spike; (8) awnless, white wheat; and (9) awned, white wheat.

**Figure 4:**
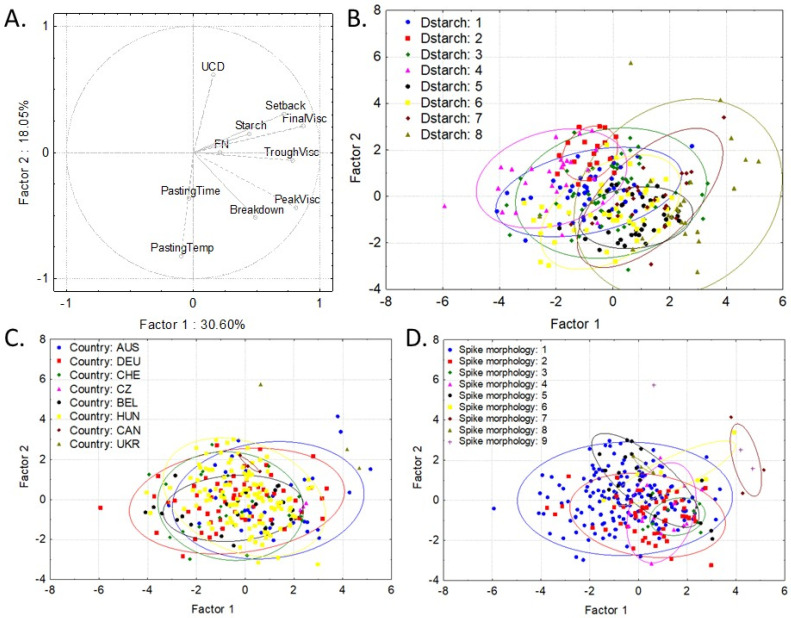
Principal component analysis based on the starch properties of spelt (**A**) and grouped by the groups formed by the hierarchical cluster analysis (**B**), country (**C**), and spike morphology (**D**) groups (where the most determinant traits that contributed to Factor 1 were peak viscosity (0.8115) and final viscosity (0.8699), while it was pasting temperature (−0.8191) and starch damage (0.6144) for Factor 2). DevTime, dough development time; FN, falling number; Temp, temperature; UCD, starch damage; Visc, viscosity; Dstarch, groups formed by starch properties on dendrogram (Figure 2b); AUS, Australia; BEL, Belgium; CAN, Canada; CZ, Switzerland; CHE, Czech Republic; DEU, Germany; HUN, Hungary; UKR, Ukraine. Spike morphology: (1) awnless, red, spelt-type spike; (2) awnless, white, spelt-type spike; (3) awned, black, spelt-type spike; (4) awned, red, spelt-type spike, (5) awned, white, spelt-type spike; (6) awnless, gray, spelt-type spike; (7) awnless, gray, wheat-type spike; (8) awnless, white wheat; and (9) awned, white wheat.

Based on the breadmaking quality traits and starch properties of the spelt genotypes, two hierarchical cluster analysis were carried out. Both of the analyses resulted in eight main groups of genotypes (Figure 2a,b). Then, in order to show the differences between the different groups and make it visually evaluable, a PCA was carried out (Figure 3 and Figure 4) based on breadmaking quality traits and starch properties, separately. Genotypes were grouped on PCA figures in three different ways: by dendrogram groups, by country of origin, and by spike morphology. Tukey’s test was also used to show the significant differences between the different groups (Supplementary Table S4).

Generally, we can say that most of the groups are overlapping, but there are some main differences that are worth highlighting. Groups 4, 5, and 6 on Figure 2a could be characterized by high dough stability and farinograph quality number, but low dough softening, while Groups 8 and 7 had the lowest dough stability and quality number and a high degree of dough softening (Figure 2a and Figure 3a,b; Supplementary Table S4a). The bread wheat varieties ‘Glenlea’ and ‘Ukrainka’ belong to Group 4, with the highest farinograph quality numbers and water absorption and the fifth and sixth highest dough stability.

No difference was found between the genotype groups originating from different countries based on their breadmaking quality (Figure 3a,c), but based on the spike morphology, Groups 3 and 6 (Group 3 = awned, black, spelt-type spike; Group 6 = awnless, gray, spelt-type spike) had higher dough stability, GI, Zeleny, and quality number than the other groups (Figure 3d and Supplementary Tables S1 and S4c,d), and the values were similar to those of wheat.

The grouping based on the starch properties (Figure 2b and Figure 4a,b) showed that Groups 5, 7, and 8 had a high peak viscosity, while Groups 2 and 4 had a low peak viscosity. At the same time, the final viscosity was high in Groups 7 and 8 and low in Groups 4 and 6. The wheat varieties belonged to different groups (Groups 6 and 8) based on their starch properties. Although they have the lowest pasting temperature and the highest starch damage (UCD), their final viscosity values were extremely different: ‘Ukrainka’ had a very high final viscosity, while it was very low in ‘Glenlea’.

The groups based on their country of origin could not be distinguished (Figure 4c), but on spike morphology, Group 7 (awnless, gray, wheat-type spike) had a higher peak and final viscosity than the other groups (Figure 4d and Supplementary Table S1), while Group 3 (awned, black, spelt-type spike) had a high final viscosity and significantly low starch damage (Supplementary Table S4c,d).

### 3.4. Characterization of Spelt Genotypes

It is important to select spelt genotypes with good kernel characteristics and breadmaking quality traits in order to efficiently use them as genetic resources in crossing programs (Supplementary Table S1).

From varieties and breeding lines bred in Martonvásár, Hungary, ’Mv Martongold’ was among those with the highest test weight, while ’Mv Vitalgold’ had high thousand kernel weight and gluten content. There are also some outstanding breeding lines originating from Martonvásár, such as ’TSP04-09′, with high protein content; ’TSP06-10′, with high gluten content; and ’TSP07-09′, with high water absorption of the flour. Younger breeding lines were also outstanding for several traits. ‘Oberkulmer/Baulander-Spelz_1′ and ‘Oberkulmer/Schwabenkorn_1′ had high protein and gluten content and water absorption, with the former having a low pasting temperature as well. ’Oberkulmer/Schwabenkorn_2′, _3, and _5′ had a high thousand kernel weight, starch damage and low pasting temperature. ’Oko-10′ was the best for thousand kernel weight, gluten content, water absorption, and pasting temperature. Several genebank accessions were also outstanding for more than four quality traits. These were the MVGB142 (high protein, GI, Zeleny, dough stability, and quality number), MVGB145 (high Zeleny, dough stability, and quality number), MVGB353 (high GI, Zeleny, and quality number), MVGB524 (high GI, Zeleny, dough stability, and quality number), and MVGB525 (high GI, Zeleny, dough stability, water absorption, and quality number).

Although ‘Mv Vitalgold’ and ‘Oko-10′ had good physical traits and gluten content, the quality of their gluten was low, resulting in higher dough softening and lower dough stability, GI, and quality number. ‘Oberkulmer/Schwabenkorn_1′,_2, and _3’ also had poor breadmaking quality, along with low Zeleny sedimentation values, dough stability, and quality number. From genebank accessions, ’MVGB525′ had low starch damage and high pasting temperature.

Regarding international cultivars, seven could be highlighted. These were the Czech ‘Bohemia’ (high GI, Zeleny, dough stability, and quality number); the German ‘Bodensonne’ and ‘D-7-014-99-02′ (with high dough stability and quality number); the Swiss ‘Ostro’ (high TKW, water absorption, starch damage, and low pasting temperature); and the Australian ‘Black-Bearded’, ‘White-Beardless’ (with high GI, Zeleny, dough stability, and quality number), and ‘Duhamelianum-Mazz’ (with high water absorption, quality number, starch damage and low pasting temperature). ‘Hercule’ and ‘CH65388′ from Switzerland, ‘Holstenkorn’ and ‘Schwabenkorn’ from Germany, or ‘Vavilovii’ and ’Albi-Spicatum’ from Australia were among the varieties with the softest kernels and doughs. The spelt variety, ‘Ostro’ also had poor breadmaking quality, along with low Zeleny, GI, dough stability, and quality number. Nevertheless, these cultivars with poor breadmaking quality could be used for different processing purposes and for the production of special and/or local food products.

## 4. Discussions

Spelt is a small-grain cereal that is available in a variety of forms in the market, such as grain, white flour, wholemeal flour, or processed products (muffins, pancake flour, assorted pasta, and prepackaged bread). Roasted spelt (called grünkern) is a flavor enhancer in foods and is used in breakfast cereals, soups, breads, and casseroles [18]. This wide usability of spelt is due to the fact that spelt genotypes have a great variation of compositional and processing quality traits.

In our study, there was great variation in all the studied traits of spelt, but the question is, which of these traits are the appropriate and useful breeding targets? According to the results, the flour yield and setback viscosity were not affected significantly by the genotypes, and the heritability of these traits was also low (0.20 and 0.51, respectively). The test weight and falling number also had low heritability values (0.51 and 0.54, respectively) compared to the other traits, so these should be excluded as breeding targets. Protein, gluten, and starch contents were genetically determined with similarly high heritability (0.77, 0.78, and 0.79, respectively), making them suitable targets for breeding, similarly to wheat. From the parameters characterizing dough quality, the GI, the Zeleny sedimentation, and the water absorption had the highest heritability (0.91, 0.86, and 0.81, respectively), while peak and trough viscosity and pasting temperature showed the highest heritability from starch properties (0.81, 0.84, and 0.83, respectively). These parameters could also be the possible targets of breeding.

It is also important to identify those traits where outstanding genotypes with extremely high or low values could be selected as being suitable for breeding purposes. Thus, if we relate the difference of the maximum and the average values to the average values in case of the abovementioned traits (Supplementary Table S1), then the maximum protein content was 11.2% higher than the average protein content of the 90 genotypes, for which the values were 21.4% for the gluten content, 6.34% for the starch content, 64.4% for the GI, 63.9% for the Zeleny sedimentation, 11.7% for the water absorption, 10.7% for the peak viscosity, 19.5% for the trough viscosity, and 3.2% for the pasting temperature. However, much higher ratios were observed for traits with lower heritability values, such as dough stability or starch damage (99.5% and 41.2%, respectively). The effect of the environment was significant on the Zeleny sedimentation, so altogether, the GI, the water absorption, and the starch damage were found to be the most appropriate target quality traits for breeding spelt.

The average protein and gluten contents of the 90 spelt varieties were significantly higher than those of wheat. This result was also found by other authors [17,37,38]. However, high protein and gluten contents were not necessarily associated with good gluten quality. The average low values of GI, Zeleny sedimentation, and farinograph dough stability of spelt all referred to a more extensible and less stable dough. Furthermore, the hydration of the flour was significantly lower, and the dough softening was higher, in spelt than in wheat. In other studies, a low GI, alveograph P-value, tenacity, and loaf volume were also found compared to common wheat [18,39,40]. Rodríguez-Quijano et al. [17] compared the quality and composition of two spelt and three bread-wheat varieties and found that spelt dough had lower strength and tenacity and higher degradation based on SDS sedimentation, alveograph, and consistograph measurements, referring to the weaker gluten of spelt.

However, in addition to the general observations on spelt wheat, observations on the properties of the individual spelt genotypes are crucial in breeding. Complex, multidimensional statistical analyses based on a multi-trait approach provide an efficient tool for the selection of breeding targets. Thus, the hierarchical cluster analysis and PCA figures were used for grouping of genotypes based on multiple traits and for the selection of individual varieties appropriate for different processing purposes, especially focusing on breadmaking. Several genebank accessions and international varieties were highlighted in the results that can be potential sources of breeding programs aimed at improving bakery properties. These are generally having high GI, Zeleny sedimentation, and/or good farinograph quality traits (namely MVGB142, MVGB145, MVGB353, MVGB524, and MVGB525; ‘Bohemia’; ‘Bodensonne’; ‘D-7-014-99-02′; ‘Black-Bearded’; ‘White-Beardless’; and ‘Duhamelianum-Mazz’). Earlier, Mikos et al. [41] also identified four spelt cultivars (Schwabenkorn, Kujawianka, Ostka Kazimierska, and Banatka Kresowa) which have satisfying breadmaking quality.

In previous studies, it was established that spelt could be characterized by a higher final pasting viscosity and pasting temperature than wheat, presumably due to the higher amylose content and B- and C-type starch granules [18]; however, a significant environmental effect was observed. As our focus is on breadmaking, we are looking for spelt genotypes with similar properties to wheat, i.e., harder kernels, with higher starch damage and water absorption of the flour, but lower pasting time and temperature. In our study, the wheat varieties (‘Glenlea’ and ‘Ukrainka’) had the lowest pasting temperature and the highest starch damage; meanwhile ‘Ukrainka’ had a high and ‘Glenlea’ had a low final viscosity. The spelt varieties with good breadmaking quality also had a low pasting temperature. From these, the ‘Duhamelianum-Mazz’ cultivar could be highlighted, with high water absorption, quality number, and starch damage and low pasting temperature at the same time.

However, further studies are necessary to determine how starch properties influence the breadmaking quality of spelt. Our correlation analysis of these traits (Supplementary Table S5) showed that starch viscosity properties (peak, trough, breakdown, final, and setback viscosity) negatively correlated with the protein and gluten content, but positively correlated with their quality determinant traits (GI, Zeleny, dough stability, and quality number). The water absorption had a positive correlation with the starch damage, but a negative correlation with the viscosity parameters and the pasting temperature. The pasting temperature also had a positive correlation with the GI, Zeleny, and dough stability. Consequently, higher viscosity values and pasting temperature come together with better gluten and dough quality in spelt.

A few studies reported on the application of spelt for breadmaking. Frakolaki et al. [42] studied the breadmaking potential of spelt flour in spelt–wheat flour mixtures (ranging from 0% to 100% spelt at 10% intervals). It has been found that a proportion of up to 20% of spelt flour results in bread-like bread quality and organoleptic characteristics, while proportions of more than 70% lead to products with poorer bread quality traits. Callejo et al. [43] found that spelt bread had high crumb elasticity, but low crumb cell homogeneity when compared to common wheat bread. Thus, the relevance of the selection of spelt genotypes with good breadmaking quality traits and their breeding is clear.

The effect of the environment is usually high on the quantitative traits, such as those included in this study. The dough softening, Zeleny sedimentation, final viscosity, and setback-viscosity were the most affected by the environment in this study; thus, they are not recommended for selection purposes. As nearly the same field management practices were applied on spelt in each year, the environmental effect is primarily attributable to the different vintages in this experiment. The effect of the environment on spelt quality was also studied before. Longin et al. [44] found that spelt had a slightly higher protein content than bread wheat, although it received a N fertilizer reduced by 35% compared to the wheat control. This clearly shows that, despite the limited N supply, spelt is able to produce a high protein content, as it was also shown by other researchers [45,46,47]. Different sowing rates had no significant effects on spelt grain quality parameters, but higher nitrogen application resulted in a decrease in the number of ears per unit area. Fertilization with N at a rate of 50 kg/ha resulted in higher starch and fat levels in spelt grains [48].

## 5. Conclusions

The physical, compositional, and breadmaking qualities of the studied spelt genotypes showed high variation in this study, thus proving that this sample set represents a good genetic base for breeding purposes, aiming to achieve the targeted specific processing traits. Some accessions and international varieties were identified which are appropriate for breadmaking quality purposes, but other specific local product requirements could also be fulfilled with the use of these genotypes.

## Figures and Tables

**Table 1:** Mean, standard deviation, and ranges of physical, compositional, and breadmaking quality traits of 90 *Triticum aestivum* subsp. *spelta* genotypes grown in 3 years in Hungary (Martonvásár, 2017–2019).

Descriptive Statistics	Mean	Standard Deviation	Minimum	Maximum	Range	‘Glenlea’	‘Ukrainka’
N = 270						*T. aestivum*	
Test weight (kg/100 L)	70.51	4.44	58.90	81.90	23.00	79.57	75.60
Thousand kernel weight (g)	36.93	5.33	23.22	49.79	26.57	42.63	38.31
Kernel width (mm)	2.93	0.17	2.50	3.50	1.00	3.24	3.18
Kernel length (mm)	7.32	0.45	6.00	8.30	2.30	7.08	6.70
Flour Yield (%)	58.22	4.31	31.82	75.12	43.30	60.17	54.72
β glucan content (mg/g)	6.54	0.66	4.53	8.46	3.93	5.95	6.79
Starch content (%)	52.94	1.46	49.20	57.80	8.60	55.73	55.57
Protein content (%)	18.99	1.61	12.17	22.20	10.03	16.30	14.53
Gluten content (%)	44.71	6.01	29.30	59.80	30.50	35.97	28.90
Gluten spread (mm/h)	7.80	3.88	1.00	20.50	19.50	2.50	1.17
Gluten Index	59.28	19.58	0.73	98.89	98.15	97.32	99.70
Zeleny sedimentation (mL)	34.78	9.67	16.00	73.00	57.00	48.00	45.67
Dough development time (min)	4.75	4.61	0.30	20.00	19.70	19.87	1.94
Dough stability (min)	9.54	6.35	0.00	19.60	19.60	17.47	18.17
Dough softening at 12 min (FU)	76.06	49.61	0.00	306.00	306.00	45.00	30.33
Water absorption (%)	55.95	2.07	51.30	63.30	12.00	62.90	60.00
Quality Number	59.73	21.29	0.00	100.00	100.00	100.00	76.90
Falling number (sec)	383.28	62.14	103.00	613.00	510.00	416.00	428.00
Peak Viscosity (cP)	3706.24	207.59	3178.00	4213.00	1035.00	3817.00	3939.00
Trough Viscosity (cP)	1751.42	110.17	1426.00	2116.00	690.00	1783.67	2072.00
Breakdown (cP)	1954.82	163.30	1622.00	2377.00	755.00	2033.33	1867.00
Final Viscosity (cP)	3824.95	276.01	2981.00	4643.00	1662.00	3528.00	4157.00
Setback (cP)	2073.53	211.28	1520.00	2965.00	1445.00	1744.33	2085.00
Pasting Time (min)	9.08	0.10	8.73	9.33	0.60	8.96	8.91
Pasting Temperature (°C)	63.48	1.01	60.90	66.00	5.10	61.60	61.17
Starch Damage (UCD)	12.75	1.79	6.30	19.40	13.10	17.35	18.85

## Data Availability

Data is contained within the article or Supplementary Material.

## Supplementary Materials

The following supporting information can be downloaded at https://www.mdpi.com/article/10.3390/foods11142061/s1. Table S1: Average of the physical, compositional, breadmaking and starch quality traits in the individual spelt genotypes grown in Hungary (Martonvásár, 2017–2019). Table S2: Growing and environmental conditions in Hungary (Martonvásár, 2017–2019). Table S3: Meteorological conditions in Hungary (Martonvásár, 2017–2019). Table S4: Mean values of the parameters for each of the dendrogram groups distinguished by quality parameters (a), starch properties (b), country of origin (c), and spike morphology (d) where the significant differences are shown by Tukey test results. Table S5: Correlation of the compositional and breadmaking quality traits with starch related properties (*n* = 90).
