# Supplementary material for: Characterization of the Protein and Carbohydrate Related Quality Traits of a Large Set of Spelt Wheat Genotypes
Source: Foods. 2022 Jul 12;11(14):2061. doi: 10.3390/foods11142061 (PMC9324691; doi:10.3390/foods11142061)
Supplement: Supplementary file 1 [file foods-11-02061-s001.zip › foods-1790226-supplementary.pdf]

Table S1: Average of the physical, compositional, breadmaking and starch quality traits in the individual spelt genotypes grown in Hungary (Martonvásár, 2017–2019).

Variety	Country	Spike morphology	Type of Spike	Colour of Spike	Form of spelt typepike	D all	D composition	D quality	D starch	Test weight	TKW	KWidth	KLength	Flour yield	Protein	Starch	B glucan	Gluten	Gluten spread	GI	Zeleny	Development Time	Dough Stability	Dough Softening at 12 min	Water absorption	Farinograph quality number	Falling Number	Peak Viscosity	Trough Viscosity	Breakdown	Final Viscosity	Setback	Pasting Time	PastingTemperature	Starch Damage
**N=92, 3-years average**		(based on combination of D-F columns)				**Dendogram groups**				**kg/100l**	**g**	**mm**	**mm**	**%**	**%**	**%**	**mg/g**	**%**	**mm**		**ml**	**min**	**min**	**FU**	**%**		**sec**	**cP**	**cP**	**cP**	**cP**	**cP**	**min**	**C**	**UCD**
ALBISPICATUM	AUS	5	AWN	WHITE	spelt type	3	3	7	2	72.80	33.90	2.75	7.20	56.52	19.95	52.50	6.12	50.83	14.00	50.33	31.00	1.70	2.00	88.00	56.75	46.85	332.00	3496.50	1745.50	1751.00	3918.50	2173.00	9.00	62.23	14.20
ALBI-SPICATUM	AUS	2	AWNLESS	WHITE	spelt type	4	5	3	3	66.25	35.72	3.00	7.27	56.71	19.45	52.00	7.25	45.22	8.67	73.27	47.00	2.13	2.87	113.67	57.80	42.30	385.00	3721.50	1875.50	1846.00	3944.00	2068.50	9.13	63.78	10.75
ALBUM	AUS	2	AWNLESS	WHITE	spelt type	8	2	5	6	71.10	37.00	2.77	7.57	53.26	18.81	52.37	6.73	47.83	8.17	61.53	34.33	9.17	13.50	43.33	57.33	75.70	423.00	3727.67	1707.00	2020.67	3641.00	1934.00	9.02	63.45	13.30
BA7415	DEU	1	AWNLESS	RED	spelt type	2	5	5	1	74.07	46.24	3.23	7.73	58.91	20.25	53.07	6.54	44.22	6.50	57.97	37.67	8.00	14.80	41.33	56.80	79.47	346.00	3629.00	1743.33	1885.67	3643.00	1899.67	9.13	63.13	13.57
BA7418	DEU	2	AWNLESS	WHITE	spelt type	7	6	2	5	75.47	38.07	2.87	7.07	61.11	17.73	55.37	5.76	37.23	6.00	66.04	29.00	7.47	8.77	53.67	54.67	65.93	406.67	3899.33	1793.00	2106.33	3844.67	2051.67	9.18	63.03	14.00
BA7423	DEU	1	AWNLESS	RED	spelt type	8	5	6	6	75.10	39.14	2.97	7.30	61.57	18.73	52.67	6.61	43.77	7.00	75.70	46.33	8.27	10.60	68.67	57.87	74.97	385.67	3782.00	1754.33	2027.67	3717.67	1963.33	9.02	62.93	11.00
BA7424	DEU	1	AWNLESS	RED	spelt type	8	7	8	6	71.77	41.46	3.10	7.53	54.97	18.12	54.33	5.99	41.15	6.50	50.38	25.67	5.90	6.23	124.67	55.50	53.83	380.67	3782.00	1699.33	2082.67	3706.00	2006.67	9.04	63.35	11.87
BADENKRONE	DEU	1	AWNLESS	RED	spelt type	7	7	5	6	60.10	32.29	3.00	7.40	55.40	16.55	52.70	6.40	40.95	7.50	41.58	30.00	0.55	6.25	70.00	53.40	27.10	327.00	3806.50	1721.00	2085.50	3792.00	2071.00	9.00	64.33	11.85
BADENSONNE	DEU	1	AWNLESS	RED	spelt type	1	4	5	1	69.40	32.03	2.90	7.45	52.69	17.17	53.10	6.59	44.75	13.25	32.34	28.50	3.35	15.85	30.50	54.35	78.50	407.50	3617.50	1723.00	1894.50	3756.50	2033.50	8.90	63.05	13.85
BALMEGG	CHE	1	AWNLESS	RED	spelt type	8	1	6	6	74.53	35.29	3.03	6.97	54.91	19.66	52.57	6.92	49.02	6.83	67.06	39.33	3.17	15.57	47.00	56.73	64.40	406.00	3825.67	1691.00	2134.67	3590.00	1899.00	9.11	63.63	13.23
BLACK-BEARDED	AUS	3	AWN	BROWN	spelt type	6	7	4	8	70.90	34.77	2.75	7.05	59.59	18.86	52.55	6.19	38.73	4.25	97.46	52.67	17.05	19.03	5.00	57.10	97.15	447.50	3880.50	1879.00	2001.50	4319.50	2440.50	9.10	65.08	11.45
BOHEMIA	CZ	3	AWN	BROWN	spelt type	6	7	4	8	71.35	35.14	2.75	7.10	60.13	18.48	52.30	6.56	38.10	4.25	92.44	52.83	14.05	18.60	5.50	55.05	88.25	485.00	3876.50	1942.50	1934.00	4272.50	2330.00	9.07	64.23	12.35
CH65384	CHE	2	AWNLESS	WHITE	spelt type	7	6	3	5	76.50	38.47	2.90	7.00	62.41	17.10	55.73	6.00	36.48	6.67	63.80	28.67	2.57	6.93	90.00	53.43	51.20	400.00	3908.00	1770.00	2138.00	3768.00	1998.00	9.13	62.80	12.57
CH65388	CHE	2	AWNLESS	WHITE	spelt type	2	2	8	1	75.93	39.63	3.03	6.83	60.23	18.54	54.80	5.97	44.65	9.50	30.93	20.33	1.87	2.43	140.33	54.53	26.63	390.33	3607.00	1685.00	1922.00	3667.00	1982.00	9.20	63.62	11.73
COSMOS	BEL	1	AWNLESS	RED	spelt type	8	7	2	6	68.03	36.63	3.00	7.17	61.69	17.79	53.53	7.39	40.18	5.67	79.53	40.00	3.30	7.57	71.67	53.10	59.10	387.67	3789.33	1681.33	2108.00	3774.67	2093.33	9.02	63.78	13.83
D-7-004-99-01	DEU	1	AWNLESS	RED	spelt type	8	6	6	6	72.67	38.01	3.00	7.30	61.61	18.21	54.63	6.65	38.47	3.83	78.26	38.00	1.97	14.97	54.33	54.03	62.93	340.33	3789.33	1710.33	2079.00	3687.00	1976.67	9.13	63.60	12.17
D-7-004-99-02	DEU	1	AWNLESS	RED	spelt type	2	5	2	1	69.90	35.95	2.93	7.30	58.73	20.10	52.17	6.62	44.50	6.33	64.54	38.33	4.60	9.43	78.67	56.33	62.47	386.00	3623.33	1664.33	1959.00	3625.00	1960.67	9.00	62.77	13.10
D-7-005-99-01	DEU	1	AWNLESS	RED	spelt type	5	3	8	4	65.73	37.49	2.97	7.83	59.22	20.83	51.53	6.66	51.97	7.00	51.98	35.00	3.33	6.83	124.33	56.90	52.83	319.00	3493.33	1669.33	1824.00	3636.67	1967.33	9.20	64.67	12.83
D-7-005-99-04	DEU	1	AWNLESS	RED	spelt type	1	5	2	1	69.83	37.39	3.03	7.30	58.70	19.45	52.27	6.79	44.65	7.17	65.85	36.67	3.57	13.93	61.67	55.90	64.27	382.00	3637.00	1727.33	1909.67	3798.33	2071.00	9.13	63.35	12.30
D-7-005-99-06	DEU	1	AWNLESS	RED	spelt type	2	5	1	1	71.07	39.42	3.03	7.40	57.37	19.96	52.83	6.64	44.77	9.17	54.41	31.33	2.40	7.20	80.33	56.37	53.70	365.67	3636.67	1670.00	1966.67	3697.67	2027.67	9.09	63.20	13.60
D-7-010-99-02	DEU	1	AWNLESS	RED	spelt type	4	7	2	3	69.77	36.28	2.83	7.53	58.41	19.41	52.97	5.62	39.02	5.50	70.52	34.00	2.70	5.73	79.67	52.90	55.27	378.00	3596.67	1763.00	1833.67	4005.00	2242.00	9.07	63.65	13.67
D-7-014-98-01	DEU	1	AWNLESS	RED	spelt type	4	5	6	3	67.13	36.10	3.03	7.37	58.33	19.38	52.23	6.35	44.73	6.50	72.81	41.00	2.53	14.93	42.33	55.97	65.73	391.00	3739.67	1772.00	1967.67	3897.67	2125.67	9.20	64.17	12.73
D-7-014-99-02	DEU	1	AWNLESS	RED	spelt type	4	5	5	3	67.90	35.57	3.00	7.33	58.77	20.08	52.53	6.76	43.65	4.83	74.13	42.00	12.60	17.40	24.33	55.00	84.37	412.33	3744.00	1753.67	1990.33	3848.67	2095.00	9.13	63.12	11.00
D-7-014-99-03	DEU	1	AWNLESS	RED	spelt type	6	6	2	7	69.37	32.89	2.90	6.97	59.05	18.96	53.00	6.71	37.38	2.50	86.57	42.67	2.67	12.17	71.00	52.90	48.93	399.33	3809.33	1829.33	1980.00	4091.33	2262.00	9.00	63.28	11.83
D-7-019-99-02	DEU	1	AWNLESS	RED	spelt type	7	4	2	5	70.50	37.43	2.90	7.50	60.02	18.20	53.60	6.93	42.68	4.67	65.99	34.00	2.97	8.00	74.67	55.67	59.33	380.33	3894.33	1700.00	2194.33	3770.33	2070.33	9.09	63.13	13.80
DUHAMELIANUM	AUS	1	AWNLESS	RED	spelt type	3	2	7	1	76.45	38.17	2.87	7.30	57.09	18.35	52.77	6.61	46.83	8.83	50.88	31.33	3.20	5.83	91.67	56.77	52.83	442.33	3522.33	1748.00	1774.33	3804.33	2056.33	9.11	62.45	13.63
DUHAMELIANUM-MAZZ	AUS	6	AWNLESS	GREY	spelt type	6	4	6	7	74.00	33.78	2.83	6.90	57.57	17.06	53.33	7.47	44.08	7.33	77.55	42.00	9.97	13.93	41.00	62.50	76.97	520.00	4104.33	1887.33	2217.00	4030.33	2143.00	8.84	61.65	18.00
EBNERS-ROTKORN	DEU	1	AWNLESS	RED	spelt type	5	3	7	4	69.70	39.07	2.95	7.60	57.13	18.45	52.40	6.95	54.25	16.25	25.08	28.00	2.60	7.60	90.50	57.15	55.65	307.50	3292.50	1535.50	1757.00	3381.50	1846.00	9.03	62.73	12.75
FRANCKENKORN	DEU	1	AWNLESS	RED	spelt type	8	7	2	6	67.93	37.81	3.00	7.53	60.98	19.20	52.77	6.70	41.13	7.00	76.69	37.67	3.07	10.90	68.67	53.90	63.33	397.00	3782.67	1668.67	2114.00	3715.67	2047.00	9.02	64.13	11.13
GEOFFS-RED-SPELT	AUS	1	AWNLESS	RED	spelt type	1	2	5	1	68.43	33.29	2.73	7.33	57.66	20.61	51.50	6.22	47.48	5.33	69.31	42.33	9.30	12.87	47.67	57.07	76.43	398.33	3623.67	1715.00	1908.67	3802.67	2087.67	9.13	64.62	12.97
GEOFFS-WHITE-SPELT	AUS	2	AWNLESS	WHITE	spelt type	7	6	6	5	73.43	32.10	2.73	6.73	57.02	17.74	54.50	7.14	35.88	4.33	87.83	33.67	9.73	15.43	51.67	53.60	74.00	392.33	3847.67	1714.33	2133.33	3724.67	2010.33	9.04	64.50	13.50
HERCULE	BEL	2	AWNLESS	WHITE	spelt type	4	2	8	3	67.90	33.03	2.87	7.10	60.69	18.30	53.03	6.21	46.45	16.50	11.86	17.00	0.80	0.73	216.67	55.60	0.00	343.00	3545.67	1594.33	1951.33	3800.00	2205.67	9.02	63.82	11.23
HOLSTENKORN	DEU	1	AWNLESS	RED	spelt type	4	1	8	3	65.70	32.03	2.73	7.27	59.10	18.58	52.70	6.93	49.02	8.17	52.60	31.33	2.30	4.20	109.67	53.93	35.03	425.00	3619.33	1653.00	1966.33	3870.00	2217.00	9.04	64.65	12.63
HUBEL	CHE	1	AWNLESS	RED	spelt type	8	7	3	6	73.77	36.12	3.00	6.97	62.45	18.05	53.93	6.29	41.35	6.33	68.25	35.67	4.03	5.73	118.67	52.80	50.83	385.00	3734.67	1673.33	2061.33	3557.00	1883.67	9.13	64.87	9.93
LUEG	CHE	1	AWNLESS	RED	spelt type	1	4	6	1	66.90	28.01	2.85	6.50	53.87	18.99	51.15	6.11	42.13	8.50	60.08	41.50	4.10	13.45	52.00	54.65	71.60	346.50	3599.00	1760.50	1838.50	3797.50	2037.00	9.10	64.93	11.85
MVGB139	HUN	2	AWNLESS	WHITE	spelt type	8	2	5	6	71.07	34.18	2.77	7.20	59.34	20.43	51.53	6.94	48.20	8.50	59.88	35.00	11.50	14.07	53.33	57.27	80.67	429.67	3850.67	1728.67	2122.00	3580.67	1852.00	9.13	63.98	12.80
MVGB140	HUN	2	AWNLESS	WHITE	spelt type	7	5	6	5	71.10	38.92	2.87	7.57	53.67	19.81	52.73	6.21	43.87	7.17	59.89	31.00	6.80	12.77	55.00	55.70	69.00	420.33	3987.00	1753.33	2233.67	3790.33	2037.00	8.98	63.47	13.27

MVGB141	HUN	4	AWN	RED	spelt type	7	2	2	5	64.00	33.19	2.73	7.30	57.12	19.57	52.47	6.48	46.87	7.50	59.85	37.00	2.55	7.50	73.00	55.80	59.65	445.00	3984.50	1893.50	2091.00	3910.50	2017.00	9.08	64.58	13.05
MVGB142	HUN	6	AWNLESS	GREY	spelt type	4	5	4	3	69.33	29.51	2.70	6.63	58.12	20.41	51.20	5.28	44.57	3.83	84.90	49.00	9.60	16.10	32.67	56.43	83.97	351.67	3621.00	1721.33	1899.67	3957.67	2236.33	8.96	63.35	13.40
MVGB143	HUN	4	AWN	RED	spelt type	4	4	6	3	63.80	29.63	2.63	7.13	55.89	19.52	52.30	6.16	42.78	6.25	65.26	39.00	2.70	11.05	44.50	55.70	64.10	439.00	3689.00	1740.50	1948.50	3923.00	2182.50	8.93	62.73	11.90
MVGB144	HUN	2	AWNLESS	WHITE	spelt type	7	7	3	5	72.10	34.72	3.03	6.53	60.90	18.85	53.60	6.46	39.52	4.00	76.78	39.00	1.50	6.17	101.00	53.50	43.23	406.33	3886.67	1749.33	2137.33	3830.67	2081.33	9.02	64.18	11.40
MVGB145	HUN	4	AWN	RED	spelt type	6	4	5	7	64.75	28.22	2.60	7.00	57.55	19.20	52.45	6.54	42.10	6.25	66.68	43.00	6.85	17.00	30.00	55.20	77.00	439.67	3865.00	1795.00	2070.00	4029.00	2234.00	9.07	63.48	13.40
MVGB146	HUN	5	AWN	WHITE	spelt type	3	2	1	2	70.07	30.88	2.60	7.13	56.12	21.11	50.97	6.03	46.07	6.50	35.14	33.33	5.50	11.73	64.33	57.33	69.47	385.33	3457.00	1744.00	1713.00	3821.50	2077.50	9.00	63.03	13.90
MVGB308	HUN	2	AWNLESS	WHITE	spelt type	6	6	8	8	75.63	41.22	3.37	6.50	62.38	16.20	56.03	7.90	33.42	7.17	27.22	26.67	2.67	4.23	125.00	54.50	42.47	269.33	3899.67	1960.33	1939.33	4153.67	2193.33	9.16	63.82	12.23
MVGB318	HUN	1	AWNLESS	RED	spelt type	6	3	7	7	68.40	37.56	3.00	7.70	56.27	19.92	52.30	6.24	50.83	7.67	46.62	30.33	1.67	4.10	104.00	57.23	46.17	340.00	3815.00	1815.67	1999.33	3974.33	2158.67	9.13	63.65	12.33
MVGB319	HUN	4	AWN	RED	spelt type	4	5	2	3	71.37	31.59	2.70	7.13	58.22	19.88	52.37	6.92	45.55	7.83	70.42	43.33	3.57	12.63	65.00	55.83	64.37	454.67	3748.67	1810.33	1938.33	3899.00	2088.67	9.20	64.80	10.17
MVGB353	HUN	1	AWNLESS	RED	spelt type	8	4	4	6	71.53	34.34	2.73	7.47	53.42	20.11	52.33	6.91	42.03	4.50	81.64	44.67	13.90	13.97	33.00	56.77	82.73	391.33	3816.33	1754.33	2062.00	3684.00	1929.67	9.07	63.80	13.53
MVGB523	HUN	2	AWNLESS	WHITE	spelt type	7	4	5	5	72.17	37.23	2.80	7.53	53.31	19.89	52.27	6.57	43.10	6.50	65.26	33.67	12.53	14.87	36.00	56.63	83.47	444.67	3849.00	1740.00	2109.00	3693.00	1953.00	9.00	63.33	14.03
MVGB524	HUN	3	AWN	BROWN	spelt type	6	4	4	7	70.85	33.01	2.75	7.05	62.15	19.54	51.85	6.31	42.83	4.00	87.78	53.50	6.95	18.85	14.00	56.75	85.25	412.50	3808.00	1876.00	1932.00	4100.50	2224.50	9.03	63.95	11.20
MVGB525	HUN	3	AWN	BROWN	spelt type	4	5	4	3	66.00	34.33	2.80	7.15	59.19	19.90	51.30	6.15	44.13	4.50	92.23	57.00	6.45	18.70	18.00	57.65	82.95	438.50	3629.00	1831.00	1798.00	4124.00	2293.00	9.17	65.50	10.10
MVGB526	HUN	5	AWN	WHITE	spelt type	3	3	1	2	72.35	33.14	2.70	7.15	59.10	20.93	51.65	6.15	53.80	10.50	48.48	35.00	8.40	10.30	74.50	57.00	69.50	381.00	3506.50	1780.50	1726.00	3989.50	2209.00	8.93	62.00	13.35
MVGB527	HUN	1	AWNLESS	RED	spelt type	4	5	6	3	70.67	36.99	2.90	7.60	58.12	19.85	52.33	6.42	45.05	5.50	67.72	41.00	8.47	14.77	50.00	56.50	73.37	381.67	3727.00	1751.67	1975.33	3828.33	2076.67	9.07	62.82	14.30
MVGB528	HUN	2	AWNLESS	WHITE	spelt type	7	5	5	7	69.63	36.32	2.77	7.63	51.13	20.46	51.57	6.45	44.32	7.83	64.15	36.67	11.83	13.07	39.33	57.40	76.87	413.67	3849.67	1768.67	2081.00	3902.00	2133.33	9.00	63.83	13.47
MVGB529	HUN	5	AWN	WHITE	spelt type	3	2	6	2	71.13	31.46	2.67	7.03	55.94	20.29	51.70	5.85	48.30	8.17	57.42	36.00	8.83	12.53	57.00	57.17	74.73	391.67	3545.67	1787.33	1758.33	3938.67	2151.33	9.00	63.02	13.57
MVGB557	HUN	2	AWNLESS	WHITE	spelt type	6	7	3	7	69.20	33.76	2.97	6.90	62.75	18.51	52.97	5.98	41.37	5.17	84.42	42.00	2.53	6.47	84.00	54.77	51.30	308.33	3814.67	1848.00	1966.67	3984.33	2136.33	9.22	63.82	10.93
MVGB855	HUN	5	AWN	WHITE	spelt type	6	6	8	7	77.20	38.40	3.10	6.40	55.27	15.86	55.30	7.08	37.95	6.50	52.61	26.33	2.57	4.40	119.67	55.27	46.07	368.33	3796.00	1853.33	1942.67	3973.33	2120.00	9.16	62.80	12.13
MV-MARTONGOLD	HUN	1	AWNLESS	RED	spelt type	5	2	6	4	77.33	42.48	3.13	7.40	57.15	18.24	54.47	5.89	46.60	10.50	54.14	31.00	3.43	12.43	49.67	57.30	65.67	371.33	3512.33	1651.00	1861.33	3532.33	1881.33	9.09	62.98	13.13
MV-VITALGOLD	HUN	1	AWNLESS	RED	spelt type	5	1	8	4	70.57	42.63	3.10	7.83	58.86	19.38	53.30	6.24	49.92	13.50	22.87	25.33	1.43	2.67	127.67	56.33	36.03	363.33	3486.33	1678.33	1808.00	3628.00	1949.67	9.16	63.47	11.97
OBERKULMER/BAULANDER-SPELZ_1	HUN	1	AWNLESS	RED	spelt type	2	3	8	1	71.33	42.54	3.03	7.93	60.98	20.57	52.27	6.24	53.82	12.50	39.94	27.67	2.50	5.17	118.00	58.93	48.20	345.33	3595.00	1717.50	1877.50	3718.50	2001.00	9.18	62.65	13.20
OBERKULMER/BAULANDER-SPELZ_2	HUN	1	AWNLESS	RED	spelt type	1	1	1	1	68.47	40.03	2.97	7.83	59.86	19.87	52.93	6.05	48.83	6.17	54.82	33.67	2.57	6.23	81.33	55.87	55.27	411.67	3548.67	1685.67	1863.00	3719.67	2034.00	9.09	63.60	12.53
OBERKULMER/ROUQUIN	HUN	1	AWNLESS	RED	spelt type	5	1	7	4	69.93	41.38	3.03	7.67	55.40	19.57	53.50	5.95	49.07	10.83	44.88	26.00	2.43	5.60	104.33	56.43	49.83	368.33	3379.33	1606.00	1773.33	3655.00	2049.00	9.09	63.17	13.53
OBERKULMER/SCHWABENKORN_1	HUN	1	AWNLESS	RED	spelt type	5	3	7	4	72.03	41.03	2.97	7.83	58.99	20.43	52.37	6.55	52.98	9.33	47.44	27.00	2.73	6.73	95.33	57.90	55.17	374.00	3470.67	1691.67	1779.00	3709.33	2017.67	9.11	62.85	12.37
OBERKULMER/SCHWABENKORN_2	HUN	1	AWNLESS	RED	spelt type	3	1	8	2	71.53	43.02	3.00	7.97	57.74	19.88	53.03	6.85	49.65	7.17	38.92	26.33	1.83	3.97	110.67	56.87	42.50	353.33	3419.33	1698.00	1721.33	3813.67	2115.67	9.13	62.47	14.37
OBERKULMER/SCHWABENKORN_3	HUN	1	AWNLESS	RED	spelt type	4	1	7	3	71.13	46.91	3.03	8.03	57.80	19.34	54.03	6.86	49.20	5.67	35.39	25.33	2.70	5.73	91.00	56.13	53.97	359.67	3592.00	1779.00	1813.00	3891.33	2112.33	9.18	62.97	14.43
OBERKULMER/SCHWABENKORN_4	HUN	1	AWNLESS	RED	spelt type	7	5	1	5	70.80	41.08	3.03	7.57	56.68	19.50	52.67	7.84	43.67	8.83	54.50	30.67	3.63	10.97	75.33	55.43	60.27	396.33	3840.67	1751.67	2089.00	3815.33	2063.67	9.09	63.15	13.13
OBERKULMER/SCHWABENKORN_5	HUN	1	AWNLESS	RED	spelt type	3	1	7	2	71.97	45.19	3.07	7.97	57.92	19.91	53.03	6.96	49.72	11.50	44.87	31.00	2.53	5.00	99.67	57.37	50.63	353.67	3418.67	1738.33	1680.33	3778.67	2040.33	9.16	61.93	13.80
OBERKULMER/SERTEL_1	HUN	1	AWNLESS	RED	spelt type	4	1	8	3	72.03	42.86	3.03	7.80	60.32	19.66	53.30	6.17	48.78	6.33	51.68	29.33	1.67	4.23	119.33	56.87	41.30	375.33	3622.00	1756.67	1865.33	3912.33	2155.67	9.09	62.95	13.60
OBERKULMER/SERTEL_2	HUN	1	AWNLESS	RED	spelt type	4	5	2	3	73.00	41.74	3.00	7.83	60.71	19.73	53.50	6.78	43.92	5.00	69.12	36.33	4.80	11.10	69.00	56.23	66.43	398.33	3566.33	1719.67	1846.67	3885.67	2166.00	9.13	63.17	13.53
OBERKULMER-ROTKORN	CHE	1	AWNLESS	RED	spelt type	5	3	7	4	72.53	45.07	3.07	7.83	60.65	19.89	53.47	6.54	54.27	9.33	39.36	28.33	2.30	5.00	99.67	57.47	51.17	319.67	3327.67	1619.33	1708.33	3566.33	1947.00	9.16	62.68	13.27
OKO-10	HUN	1	AWNLESS	RED	spelt type	5	3	8	4	71.67	43.37	3.00	7.73	56.43	18.77	53.40	6.95	52.87	14.33	26.30	24.33	1.80	2.90	118.33	57.53	41.23	311.33	3347.67	1616.67	1731.00	3599.00	1982.33	9.11	62.00	13.80
OSTAR	CHE	1	AWNLESS	RED	spelt type	4	5	7	3	69.53	42.01	3.13	7.80	56.68	19.39	52.83	6.75	44.45	7.50	47.20	30.67	3.30	5.47	86.00	54.63	48.07	336.00	3688.00	1829.67	1858.33	3926.67	2097.00	9.16	63.10	13.00
OSTRO	CHE	1	AWNLESS	RED	spelt type	5	1	8	3	71.60	44.49	3.00	7.90	58.52	19.37	52.97	6.76	49.12	13.67	25.27	23.00	2.57	3.93	125.00	57.70	44.47	353.33	3436.33	1704.00	1732.33	3630.67	1926.67	9.13	62.13	13.97
POEME	BEL	1	AWNLESS	RED	spelt type	2	5	6	1	69.43	35.16	2.97	7.37	62.61	19.04	52.17	6.99	45.18	8.83	56.99	35.00	4.47	14.53	49.00	54.70	70.10	385.67	3605.00	1642.00	1963.00	3665.67	2023.67	9.09	63.35	12.10
RED WINTER	AUS	1	AWNLESS	RED	spelt type	1	5	6	1	69.60	33.00	2.77	7.37	56.11	19.72	51.93	6.44	45.85	6.33	71.98	41.00	6.87	14.47	51.33	56.57	74.37	457.00	3612.33	1699.67	1912.67	3735.67	2036.00	9.11	63.97	12.10
REDOUTE	BEL	2	AWNLESS	WHITE	spelt type	5	3	8	4	63.53	32.07	2.83	7.27	57.03	20.26	50.87	6.82	51.50	14.17	32.53	32.00	2.53	3.87	125.33	57.53	33.30	390.00	3460.00	1657.67	1802.33	3545.33	1887.67	8.98	63.35	12.63
RESSAC	BEL	1	AWNLESS	RED	spelt type	7	4	1	5	68.00	40.57	3.23	7.57	58.55	17.66	53.50	6.22	44.27	11.00	49.78	29.67	3.40	9.73	78.00	55.20	58.03	349.00	3879.00	1771.67	2107.33	3818.67	2047.00	9.11	63.78	9.63
ROUQUIN	BEL	1	AWNLESS	RED	spelt type	8	4	6	8	66.25	36.75	2.97	7.57	58.52	19.70	52.00	6.86	43.42	5.83	78.81	39.33	4.33	12.80	51.67	53.83	69.67	351.33	3677.50	1652.00	2025.50	3738.00	2086.00	9.00	64.48	14.70

SAHARENSE	AUS	7	AWNLESS	GREY	wheat type	6	6	3	8	72.07	32.31	2.87	6.20	62.23	14.87	54.53	6.75	38.65	10.50	69.63	28.00	4.63	6.50	88.33	58.67	58.77	416.00	4009.67	2075.67	1934.00	4533.00	2457.33	8.93	62.10	14.83
SCHWABENKORN	DEU	1	AWNLESS	RED	spelt type	4	6	1	3	70.53	36.77	3.00	6.97	60.93	16.25	55.13	6.95	39.53	10.00	49.71	26.33	2.67	4.37	73.67	53.27	56.93	383.00	3670.00	1751.67	1918.33	3893.67	2142.00	9.07	64.30	14.83
SERTEL	CHE	2	AWNLESS	WHITE	spelt type	7	6	3	5	76.37	38.52	2.93	7.00	63.14	17.39	55.43	5.65	39.02	7.17	73.04	31.00	2.73	5.77	85.00	53.10	51.40	389.00	3933.67	1776.33	2157.33	3733.67	1957.33	9.18	62.98	11.73
SPY	BEL	1	AWNLESS	RED	spelt type	7	4	1	5	68.00	39.52	3.20	7.53	56.87	17.97	53.40	6.07	44.40	7.17	53.71	29.00	7.97	9.90	73.67	55.17	63.33	343.33	3918.67	1797.67	2121.00	3877.33	2079.67	9.09	63.80	11.97
STONE	BEL	1	AWNLESS	RED	spelt type	8	5	6	6	68.10	37.50	3.07	7.33	57.60	19.31	52.10	6.92	44.18	7.83	58.43	37.00	4.67	13.23	60.33	55.77	66.57	412.33	3743.50	1653.00	2090.50	3720.50	2067.50	9.10	64.00	13.15
TSP04-09	HUN	1	AWNLESS	RED	spelt type	8	2	2	3	69.47	35.49	2.90	7.53	56.36	20.66	51.57	7.14	46.90	7.33	63.27	32.67	4.50	10.97	64.00	56.73	65.30	407.67	3715.67	1705.00	2010.67	3782.33	2077.33	9.09	63.82	13.50
TSP06-10	HUN	1	AWNLESS	RED	spelt type	1	1	7	1	70.93	40.84	3.03	7.67	58.78	19.49	52.83	6.61	49.73	11.83	35.59	27.00	2.67	7.03	96.33	56.87	50.50	416.33	3624.00	1735.67	1888.33	3829.00	2093.33	9.09	62.80	12.50
TSP07-09	HUN	1	AWNLESS	RED	spelt type	6	1	7	6	68.20	36.56	2.97	7.70	56.54	20.03	52.23	6.58	48.88	10.33	39.99	30.00	2.27	7.73	86.67	57.37	54.27	361.67	3871.00	1828.33	2042.67	3947.33	2119.00	9.13	63.80	13.10
VAVILOVII	AUS	5	AWN	WHITE	spelt type	6	6	8	8	75.20	35.33	3.07	6.40	53.31	14.51	56.30	6.11	33.23	9.25	57.25	29.67	1.80	4.55	127.00	54.25	39.90	251.33	3979.50	2092.50	1887.00	4259.00	2166.50	9.27	64.78	10.90
VULPINUM ALEF.	AUS	4	AWN	RED	spelt type	6	7	3	6	73.90	44.79	3.10	7.70	58.45	16.96	54.20	6.48	41.23	6.83	73.79	41.00	3.30	9.83	80.67	56.63	61.83	347.33	3894.67	1892.00	2002.67	3942.33	2050.33	9.16	63.83	13.97
WEIHENSTEPHAN	HUN	2	AWNLESS	WHITE	spelt type	6	4	8	8	69.50	32.98	2.97	6.60	60.99	17.64	53.53	6.51	43.02	10.33	61.51	36.00	2.10	5.47	128.00	55.37	34.73	286.00	3955.67	1958.67	1997.00	4086.00	2127.33	9.22	64.32	7.60
WHITE BEARDLESS	AUS	2	AWNLESS	WHITE	spelt type	7	6	4	5	72.77	32.03	2.73	6.77	57.56	18.10	53.97	7.35	37.25	2.67	91.81	39.67	7.63	16.00	29.67	54.43	80.67	398.67	4011.33	1753.67	2257.67	3714.67	1961.00	9.02	64.70	13.50
ZOLLERNSPELZ	DEU	1	AWNLESS	RED	spelt type	2	3	1	1	64.95	33.42	2.85	7.50	59.34	18.36	51.40	6.33	50.50	5.50	60.93	40.50	2.45	7.25	80.00	56.70	51.90	425.00	3618.50	1618.50	2000.00	3656.50	2038.00	8.93	62.38	12.65
GLENLEA	CAN	8	AWNLESS	WHITE	wheat type	8	6	4	6	79.57	42.63	3.24	7.08	60.17	16.30	55.73	5.95	35.97	2.50	97.32	48.00	19.87	17.47	45.00	62.90	100.00	416.00	3817.00	1783.67	2033.33	3528.00	1744.33	8.96	61.60	17.35
UKRAINKA	UKR	9	AWN	WHITE	wheat type	6	6	4	8	75.60	38.31	3.18	6.70	54.72	14.53	55.57	6.79	28.90	1.17	99.70	45.67	1.94	18.17	30.33	60.00	76.90	428.00	3939.00	2072.00	1867.00	4157.00	2085.00	8.91	61.17	18.85
**mean of spelt**										**70.51**	**36.93**	**2.93**	**7.32**	**58.22**	**18.99**	**52.94**	**6.54**	**44.71**	**7.80**	**59.28**	**34.78**	**4.75**	**9.54**	**76.06**	**55.95**	**59.73**	**383.28**	**3706.24**	**1751.42**	**1954.81**	**3824.95**	**2073.53**	**9.08**	**63.48**	**12.75**
**sd of spelt**										**3.28**	**4.25**	**0.15**	**0.41**	**2.58**	**1.32**	**1.16**	**0.47**	**4.78**	**2.95**	**18.08**	**7.54**	**3.46**	**4.71**	**35.24**	**1.62**	**16.22**	**44.64**	**177.15**	**95.24**	**137.52**	**187.53**	**116.40**	**0.08**	**0.80**	**1.38**
**min of spelt**										**60.10**	**28.01**	**2.60**	**6.20**	**51.13**	**14.51**	**50.87**	**5.28**	**33.23**	**2.50**	**11.86**	**17.00**	**0.55**	**0.73**	**5.00**	**52.80**	**0.00**	**251.33**	**3292.50**	**1535.50**	**1680.33**	**3381.50**	**1846.00**	**8.84**	**61.65**	**7.60**
**max of spelt**										**77.33**	**46.91**	**3.37**	**8.03**	**63.14**	**21.11**	**56.30**	**7.90**	**54.27**	**16.50**	**97.46**	**57.00**	**17.05**	**19.03**	**216.67**	**62.50**	**97.15**	**520.00**	**4104.33**	**2092.50**	**2257.67**	**4533.00**	**2457.33**	**9.27**	**65.50**	**18.00**
**max-avg/avg**										9.68	27.01	15.00	9.81	8.46	11.19	6.34	20.79	21.38	111.61	64.40	63.89	258.91	99.49	184.87	11.70	62.64	35.67	10.74	19.47	15.49	18.51	18.51	2.02	3.18	41.15

Blue - winter wheat

Table S2. Growing and environmental conditions in Hungary (Martonvásár, 2017-2019)

Growing conditions		2017, 2018, 2019
**Location**	geographic coordinates	47º18′N, 18º47′E
	altitude	115 m
**Growing parameters**	previous crop:	facelia, oil radish, facelia
	sowing density	280 seeds/m^2
**Soil parameters**	soil type	chernozem
	pH (KCl)	7.25
	humus (m/m%)	2.8
	P_2O_5 (mg/kg)	210
	K_2O (mg/kg)	210
	yearly average N input through NPK combined fertilizer (active ingredient, kg/ha)	120

Table S3. Meteorological conditions in Hungary (Martonvásár, 2017-2019)

		2016/2017	2017/2018	2018/2019
full season	Growing period (days)	288	282	279
	Cumulative precipitation (mm)	254.7	494.5	365.6
	Mean temperature (°C)	8.3	9.67	9.3
	Absolute min temperature (°C)	-20.7	-20.2	-14.4
	Absolute max temperature (°C)	35.6	32	36.0
last 100 days	Cum. precipitation before harvest (mm)	125.3	189.7	225.0
	Mean temperature (°C)	17.1	19.39	17.1
	Absolute min temp(°C)	-0.6	2.6	-0.7
	Absolute max temp (°C)	35.6	32.0	36.0
abs. min-max	No of days with Tmin ≤ 0 °C	95	82	90
	No of days with Tmin ≤ -10 °C	14	3	6
	No of days with Tmax ≥ 25 °C	49	68	42
	No of days with Tmax ≥ 30 °C	17	8	16
	No of days with Tmax ≥ 35 °C	1	0	1

Table S4. Mean values of the parameters for each of the dendogram groups distinguished by quality parameters (a), starch properties (b), country of origin (c.) and spike morphology (d.) where the significant differences are shown by Tukey test results.

**a.Dendogram quality group**	**1**	**2**	**3**	**4**	**5**	**6**	**7**	**8**
**N**	**27**	**36**	**24**	**27**	**30**	**48**	**36**	**48**
Test weight (kg/100L)	69.36^{a}	69.89^{a}	72.52^{a}	71.99^{a}	68.86^{a}	70.70^{a}	69.47^{a}	71.05^{a}
TKW (g)	37.20^{abc}	36.20 ^{ab}	36.80 ^{ab}	34.89 ^{a}	35.24 ^{ab}	35.27 ^{ab}	40.64 ^{c}	38.91^{bc}
KWidth (mm)	2.96^{ab}	2.90 ^{ab}	2.98 ^{ab}	2.85^{a}	2.86^{a}	2.90 ^{ab}	2.99^{b}	3.01^{b}
KLength (mm)	7.41^{bc}	7.35^{b}	6.95^{a}	7.00^{a}	7.42^{bc}	7.21^{ab}	7.69^{c}	7.31^{b}
FY (%)	58.32^{abc}	59.26^{bc}	61.13^{c}	58.34^{abc}	55.80^{a}	57.53^{ab}	57.48^{ab}	58.50^{abc}
B glucan (mg/g)	6.47^{a}	6.65^{a}	6.36^{a}	6.39^{a}	6.57^{a}	6.56^{a}	6.56^{a}	6.59^{a}
Starch (%)	52.72^{ab}	52.96^{abc}	54.05^{d}	52.98^{abc}	52.31^{a}	52.68^{ab}	52.86^{ab}	53.54^{cd}
Protein (%)	19.07^{b}	19.22^{b}	17.65^{a}	18.47^{ab}	19.34^{b}	19.08^{b}	19.56^{b}	18.53^{ab}
Gluten (%)	46.20^{cd}	42.50^{abc}	40.35^{ab}	39.17^{a}	44.66^{c}	43.96^{bc}	50.09^{d}	46.03^{cd}
Gluten sp (mm)	8.31^{bc}	6.04^{ab}	6.92^{b}	3.52^{a}	7.47^{b}	6.92^{b}	10.26^{c}	10.16^{c}
GI	51.28^{bc}	69.87^{e}	72.87^{e}	91.70^{f}	59.28^{cd}	68.12^{de}	42.30^{ab}	39.62^{a}
Zeleny (ml)	32.17^{ab}	36.81^{bc}	36.54^{bc}	49.22^{d}	36.32^{bc}	38.26^{c}	28.83^{a}	27.25^{a}
DevTime (min)	4.33^{ab}	3.81ab	2.93^{ab}	10.83^{d}	8.57^{cd}	5.64^{bc}	2.51^{a}	2.35^{a}
Stability (min)	8.63^{bc}	9.89^{c}	6.28^{ab}	17.43^{e}	13.97^{de}	13.59^{d}	5.65^{ab}	4.11^{a}
Soft at 12 (FU)	75.69^{cd}	69.22^{bcd}	95.17^{d}	23.69^{a}	41.58^{ab}	51.59^{abc}	94.43^{d}	128.73^{e}
Wabs (%)	55.81^{ab}	55.00^{a}	55.09^{a}	57.45^{c}	56.05^{abc}	56.16^{abc}	56.84^{bc}	56.10^{ab}c
HunQN	59.82^{bc}	61.20^{bc}	51.36^{b}	86.43^{e}	73.96^{d}	69.89^{cd}	51.26^{b}	38.66^{a}
FN (sec)	382.2^{abc}	401.8^{cd}	379.6^{abc}	418.8^{d}	404.1^{d}	399.5^{cd}	359.2^{ab}	345.3^{a}
PeakVisc (cP)	3675.0^{ab}	3753.9^{bc}	3862.9^{c}	3822.0^{c}	3756.2^{bc}	3736.6^{bc}	3541.4^{a}	3627.8^{ab}
TroughVisc (cP)	1730.1^{a}	1746.2^{a}	1832.5^{b}	1845.9^{b}	1739.5^{a}	1726.2^{a}	1722.7^{a}	1749.6^{a}
Breakdown (cP)	1944.9^{bc}	2007.6^{c}	2030.4^{c}	1976.1^{bc}	2016.7^{c}	2010.4^{c}	1818.7^{a}	1878.1^{ab}
FinalVisc (cP)	3809.9^{ab}	3841.8^{ab}	3911.6^{ab}	3984.2^{b}	3768.8^{a}	3769.8^{a}	3781.8^{ab}	3812.4^{ab}
Setback (cP)	2079.8^{a}	2095.5^{a}	2079.1^{a}	2138.2^{a}	2029.3^{a}	2043.5^{a}	2059.1^{a}	2062.7^{a}
PastingTime (min)	9.04^{ab}	9.08^{abc}	9.11^{cd}	9.03^{a}	9.05^{ab}	9.05^{ab}	9.11^{cd}	9.13^{d}
PastingTemp (°C)	63.25^{ab}	63.62^{ab}	63.54^{ab}	63.71^{b}	63.63^{ab}	63.51^{ab}	62.86^{a}	63.43^{ab}
Starch damage (UCD)	12.84^{ab}	12.83^{ab}	12.01^{a}	13.53^{b}	13.02^{ab}	13.17^{ab}	13.24^{ab}	12.29^{ab}

DevTime- Farinograph dough development time, FN- falling number, FY-flour yield, GI –gluten index, Gluten sp- gluten spread, HunQN- Farinograph quality number, KLength-kernel length, KWidth- kernel width, Soft at 12- dough softening at 12 min, Stability- Farinograph dough stability, Temp-Temperature, TKW- thousand kernel weight, Visc- Viscosity, Wabs- water absorption,

**b.Dendogram starch group**	**1**	**2**	**3**	**4**	**5**	**6**	**7**	**8**
**N**	**45**	**18**	**54**	**27**	**39**	**45**	**24**	**24**
Test weight (kg/100L)	70.45^{a}	71.64^{a}	67.95^{a}	70.34^{a}	71.63^{a}	71.16^{a}	70.43^{a}	72.06^{a}
TKW (g)	37.01^{ab}	36.27^{a}	36.72^{ab}	40.51^{b}	37.06^{ab}	37.58^{ab}	34.24^{a}	35.85^{a}
KWidth (mm)	2.95^{bc}	2.80^{a}	2.91^{abc}	3.01^{c}	2.92^{abc}	2.98^{bc}	2.86^{ab}	2.99^{bc}

KLength (mm)	7.39^{cd}	7.41^{cd}	7.41^{cd}	7.67^{d}	7.21^{bc}	7.35^{bc}	7.07^{ab}	6.76^{a}
FY (%)	58.20^{a}	57.23^{a}	58.49^{a}	57.87^{a}	58.34^{a}	58.16^{a}	57.72^{a}	58.98^{a}
B glucan (mg/g)	6.45^{a}	6.32^{a}	6.52^{a}	6.50^{a}	6.51^{a}	6.63^{a}	6.60^{a}	6.71^{a}
Starch (%)	52.48^{a}	52.15^{a}	52.68^{ab}	52.81^{ab}	53.79^{bc}	53.18^{ab}c	52.85^{ab}	54.10^{c}
Protein (%)	19.36^{bc}	20.34^{c}	19.40^{bc}	19.54^{bc}	18.42^{b}	18.55^{b}	18.69^{b}	16.85^{a}
Gluten (%)	46.53^{cd}	49.73^{de}	45.11^{c}	51.49^{e}	41.09^{ab}	42.96^{bc}	42.61^{bc}	37.18^{a}
Gluten sp (mm)	8.38^{ab}	9.64^{bc}	7.42^{ab}	11.69^{c}	6.44^{a}	6.62^{a}	5.91^{a}	6.59^{a}
GI	53.77^{bc}	45.86^{ab}	59.85^{cd}	38.29^{a}	66.79^{cd}	67.33^{cd}	70.80^{d}	73.00^{d}
Zeleny (ml)	34.19^{abc}	32.11^{ab}	35.85^{abc}	28.56^{a}	32.77^{abc}	37.51^{bc}	39.56^{c}	38.85^{bc}
DevTime (min)	4.13^{ab}	4.80^{ab}	4.23^{ab}	2.51^{a}	5.50^{ab}	6.33^{b}	5.63^{ab}	6.07^{ab}
Stability (min)	10.03^{ab}	7.59^{ab}	9.16^{ab}	5.96^{a}	10.22^{ab}	11.17^{b}	11.25^{b}	11.17^{b}
Soft at 12 (FU)	73.34^{ab}	82.36^{ab}	79.14^{ab}	103.91^{b}	67.44^{ab}	68.40^{ab}	62.88^{a}	70.10^{ab}
Wabs (%)	56.16^{ab}	57.08^{b}	55.83^{ab}	57.17^{b}	54.79^{a}	56.09^{ab}	56.50^{b}	56.10^{ab}
HunQN	61.08^{a}	58.95^{a}	57.11^{a}	48.99^{a}	63.04^{a}	65.22^{a}	63.57^{a}	63.48^{a}
FN (sec)	393.7^{ab}	366.1^{ab}	389.6^{ab}	347.1^{a}	397.8^{b}	386.0^{ab}	400.2^{b}	366.8^{ab}
PeakVisc (cP)	3606.6^{b}	3473.9^{a}	3648.4^{b}	3418.8^{a}	3910.7^{d}	3800.8^{c}	3857.7^{cd}	3902.2^{cd}
TroughVisc (cP)	1702.3^{ab}	1748.9^{b}	1750.6^{b}	1636.1^{a}	1766.5^{b}	1729.7^{b}	1834.1^{c}	1954.0^{d}
Breakdown (cP)	1904.2^{b}	1725.0^{a}	1897.7^{b}	1782.7^{a}	2144.2^{f}	2071.1^{de}	2023.5^{cd}	1948.1^{bc}
FinalVisc (cP)	3727.8^{abc}	3876.7^{bcd}	3890.0^{cd}	3583.7^{a}	3791.6^{bc}	3705.5^{ab}	4010.6^{de}	4189.8^{e}
Setback (cP)	2025.4^{abc}	2127.8^{bcd}	2139.3^{cd}	1947.5^{a}	2025.1^{abc}	1975.8^{ab}	2176.4^{cd}	2235.7^{d}
PastingTime (min)	9.09^{a}	9.04^{a}	9.10^{a}	9.10^{a}	9.08^{a}	9.07^{a}	9.06^{a}	9.08^{a}
PastingTemp (°C)	63.32^{b}	62.44^{a}	63.60^{b}	63.10^{ab}	63.65^{b}	63.67^{b}	63.31^{b}	63.74^{b}
Starch damage (UCD)	12.78^{a}	13.86^{a}	12.71^{a}	12.92^{a}	12.74^{a}	12.81^{a}	12.91^{a}	12.86^{a}

**c. Country**	**1**	**2**	**3**	**4**	**5**	**6**	**7**	**8**
**N**	**39**	**63**	**27**	**3**	**24**	**114**	**3**	**3**
Test weight (kg/100L)	72.07^{ab}	69.46^{ab}	73.07^{abc}	71.35^{ab}	67.41^{a}	70.59^{ab}	79.57^{c}	75.60^{bc}
TKW (g)	35.09^{a}	36.90^{a}	38.62^{a}	35.14^{a}	36.40^{a}	37.33^{a}	42.63^{a}	38.31^{a}
KWidth (mm)	2.84^{a}	2.96^{ab}	2.99^{abc}	2.75^{a}	3.02^{abc}	2.91^{a}	3.24^{c}	3.18^{bc}
KLength (mm)	7.06^{a}	7.39^{a}	7.20^{a}	7.10^{a}	7.36^{a}	7.39^{a}	7.08^{a}	6.70^{a}
FY (%)	57.16^{a}	58.68^{a}	59.21^{a}	60.13^{a}	59.20^{a}	57.83^{a}	60.17^{a}	54.72^{a}
B glucan (mg/g)	6.68^{a}	6.55^{a}	6.33^{a}	6.56^{a}	6.69^{a}	6.50^{a}	5.95^{a}	6.79^{a}
Starch (%)	53.27^{ab}	53.02^{a}	53.65^{abc}	52.30^{a}	52.58^{a}	52.72^{a}	55.73^{c}	55.57^{bc}
Protein (%)	18.08^{bc}	18.76^{bc}	18.71^{bc}	18.48^{bc}	18.75^{bc}	19.55^{c}	16.30^{ab}	14.53^{a}
Gluten (%)	42.55^{bc}	43.73^{bc}	44.50^{bc}	38.10^{abc}	44.95^{c}	46.16^{bc}	35.97^{ab}	28.90^{a}
Gluten sp (mm)	7.42^{bc}	7.20^{abc}	8.39^{bc}	4.25^{abc}	9.63^{c}	7.82^{bc}	2.50^{ab}	1.17^{a}
GI	71.74^{ab}	60.67^{a}	52.78^{a}	92.44^{b}	52.71^{a}	56.30^{a}	97.32^{b}	99.70^{b}
Zeleny (ml)	37.97^{abc}	34.95^{ab}	30.94^{a}	52.83^{c}	32.38^{a}	34.53^{ab}	48.00^{bc}	45.67^{abc}
DevTime (min)	6.65^{a}	4.09^{a}	2.96^{a}	14.05^{b}	3.93^{a}	4.81^{a}	19.87^{b}	1.94^{a}
Stability (min)	10.52^{ab}	9.88^{ab}	7.14^{a}	18.60^{b}	9.05^{ab}	9.45^{ab}	17.47^{b}	18.17^{b}
Soft at 12 (FU)	66.08^{ab}	71.56^{ab}	93.74^{b}	5.50^{a}	90.79^{b}	76.53^{ab}	45.00^{ab}	30.33^{ab}
Wabs (%)	56.88^{ab}	55.21^{a}	55.01^{a}	55.05^{a}	55.11^{a}	56.47^{a}	62.90^{c}	60.00^{bc}
HunQN	65.98^{ab}	59.64^{ab}	51.09^{a}	88.25^{bc}	52.51^{a}	60.46^{ab}	100.00^{c}	76.90^{abc}
FN (sec)	400.8^{ab}	378.3^{a}	369.5^{a}	485.0^{b}	370.2^{a}	383.3^{a}	416.0^{ab}	428.0^{ab}
PeakVisc (cP)	3802.4^{a}	3688.7^{a}	3673.3^{a}	3876.5^{a}	3702.3^{a}	3687.1^{a}	3817.0^{a}	3939.0^{a}
TroughVisc (cP)	1829.6^{ab}	1710.5^{a}	1723.2^{a}	1942.5^{bc}	1681.2^{a}	1763.7^{a}	1783.6^{ab}	2072.0^{c}

Breakdown (cP)	1972.8^{a}	1978.2^{a}	1950.0^{a}	1934.0^{a}	2021.1 ^{a}	1923.4 ^{a}	2033.3 ^{a}	1867.0 ^{a}
FinalVisc (cP)	3951.5^{abc}	3763.5^{ab}	3692.9^{a}	4272.5^{c}	3742.5^{ab}	3852.4^{abc}	3528.0^{a}	4157.0^{bc}
Setback (cP)	2121.8^{bc}	2053.0^{abc}	1969.7^{ab}	2330.0^{c}	2061.3^{abc}	2088.7^{bc}	1744.3^{a}	2085.0^{bc}
PastingTime (min)	9.07^{abc}	9.07^{abc}	9.14^{c}	9.07^{abc}	9.05^{abc}	9.09^{bc}	8.96^{ab}	8.91^{a}
PastingTemp (°C)	63.62^{b}	63.47^{b}	63.42^{b}	64.23^{b}	63.79^{b}	63.37^{b}	61.60^{a}	61.17^{a}
Starch damage (UCD)	13.32^{a}	12.72^{a}	12.36^{a}	12.35^{a}	12.41^{a}	12.75^{a}	17.35^{b}	18.85^{b}

**d. Spike morphology**	**1**	**2**	**3**	**4**	**5**	**6**	**7**	**8**	**9**
**N**	**162**	**54**	**12**	**15**	**18**	**6**	**3**	**3**	**3**
Test weight (kg/100L)	70.10^{ab}	71.65^{ab}	69.78^{ab}	67.56^{a}	73.13^{abc}	71.67^{ab}	72.07^{abc}	79.57c	75.60^{bc}
TKW (g)	38.41^{ab}	35.89^{ab}	34.31^{ab}	33.48^{a}	33.85^{a}	31.64^{a}	32.31^{a}	42.63^{b}	38.31^{ab}
KWidth (mm)	2.98^{ab}	2.90a	2.76^{a}	2.75^{a}	2.81^{a}	2.77^{a}	2.87^{a}	3.24^{b}	3.18^{b}
KLength (mm)	7.51^{c}	7.06^{bc}	7.09^{bc}	7.25^{bc}	6.89^{b}	6.77^{ab}	6.20^{a}	7.08^{bc}	6.70^{ab}
FY (%)	58.21^{a}	58.54 ^{a}	60.27 ^{a}	57.45 ^{a}	56.04 ^{a}	57.84 ^{a}	62.23 ^{a}	60.17 ^{a}	54.72 ^{a}
B glucan (mg/g)	6.59 ^{a}	6.55 ^{a}	6.30 ^{a}	6.51 ^{a}	6.22 ^{a}	6.38 ^{a}	6.75 ^{a}	5.95 ^{a}	6.79 ^{a}
Starch (%)	52.84 ^{ab}	53.46^{abc}	52.00^{a}	52.76ab	53.07^{ab}	52.27^{ab}	54.53^{bc}	55.73c	55.57^{c}
Protein (%)	19.20^{c}	18.62^{bc}	19.19^{c}	19.03c	18.77^{bc}	18.73^{bc}	14.87^{a}	16.30^{ab}	14.53^{a}
Gluten (%)	46.03^{c}	42.13^{bc}	40.94^{bc}	43.71^{bc}	45.03^{bc}	44.33^{bc}	38.65^{abc}	35.97^{ab}	28.90^{a}
Gluten sp (mm)	8.02^{bc}	7.81^{bc}	4.25^{abc}	6.93^{abc}	9.15^{c}	5.58^{abc}	10.50^{c}	2.50^{ab}	1.17^{a}
GI	55.64^{ab}	60.65^{ab}	92.48^{cd}	67.20^{abc}	50.20^{a}	81.23^{bcd}	69.63^{abcd}	97.32^{cd}	99.70^{d}
Zeleny (ml)	33.48^{abc}	32.93^{ab}	54.00^{cd}	40.67^{abcd}	31.89^{ab}	45.50^{bcd}	28.00^{a}	48.00^{cd}	45.67^{bcd}
DevTime (min)	3.94^{ab}	5.45^{ab}	11.13^{b}	3.79^{ab}	4.80^{ab}	9.78^{b}	4.63ab	19.87c	1.94^{a}
Stability (min)	9.07^{abc}	8.52^{abc}	18.80^{c}	11.60^{abc}	7.59^{ab}	15.02^{abc}	6.50^{a}	17.47^{bc}	18.17^{c}
Soft at 12 (FU)	78.63^{a}	87.28 ^{a}	10.63 ^{a}	58.63 ^{a}	88.42 ^{a}	36.83 ^{a}	88.33 ^{a}	45.00 ^{a}	30.33 ^{a}
Wabs (%)	55.88^{a}	55.40^{a}	56.64^{abc}	55.83^{a}	56.29^{ab}	59.47^{bcd}	58.67^{abc}	62.90^{d}	60.00^{cd}
HunQN	58.27^{a}	54.60^{a}	88.40^{ab}	65.39^{ab}	57.75^{a}	80.47^{ab}	58.77^{a}	100.00^{b}	76.90^{ab}
FN (sec)	375.8 ^{a}	383.1 ^{a}	445.9 ^{a}	425.1 ^{a}	351.6 ^{a}	435.8 ^{a}	416.0 ^{a}	416.0 ^{a}	428.0 ^{a}
PeakVisc (cP)	3646.6^{a}	3819.7^{ab}	3798.5^{ab}	3836.4^{ab}	3630.2^{a}	3862.7^{ab}	4009.7^{b}	3817.0^{ab}	3939.0^{ab}
TroughVisc (cP)	1712.0^{a}	1768.5^{ab}	1882.1^{b}	1826.3^{ab}	1833.9^{ab}	1804.3^{ab}	2075.7^{c}	1783.7^{ab}	2072.0^{c}
Breakdown (cP)	1934.6 ^{a}	2051.2 ^{a}	1916.4 ^{a}	2010.1 ^{a}	1796.3 ^{a}	2058.3 ^{a}	1934.0 ^{a}	2033.3 ^{a}	1867.0 ^{a}
FinalVisc (cP)	3757.4^{ab}	3800.2^{abc}	4204.1^{cd}	3940.8^{abc}	3983.4^{bc}	3994.0^{bc}	4533.0^{d}	3528.0^{a}	4157.0^{bcd}
Setback (cP)	2045.4^{ab}	2031.7^{ab}	2322.0^{bc}	2114.5^{bc}	2149.6^{bc}	2189.7^{bc}	2457.3^{c}	1744.3^{a}	2085.0^{ab}
PastingTime (min)	9.09^{b}	9.09^{b}	9.09^{b}	9.09^{b}	9.06^{ab}	8.90^{a}	8.93^{ab}	8.96^{ab}	8.91^{a}
PastingTemp (°C)	63.40^{cde}	63.71^{cde}	64.69^{e}	63.88^{de}	62.97^{bcd}	62.50^{abcd}	62.10^{abc}	61.60^{ab}	61.17^{a}
Starch damage (UCD)	12.87^{abc}	12.26^{ab}	11.28^{a}	12.50^{ab}	13.01^{abc}	15.70^{cd}	14.83^{bcd}	17.35^{de}	18.85^{e}

Table S5. Correlation of the compositional and breadmaking quality traits with starch related properties (n=90)

	Falling Number	Peak Viscosity	Trough Viscosity	Breakdown Viscosity	Final Viscosity	Setback Viscosity	Pasting Time	PastingTemperature	Starch Damage
Protein content	0.1753	-0.4648	-0.4297	-0.3011	-0.3236	-0.1697	-0.0283	-0.0582	0.0829
Starch content	-0.2877	0.3236	0.2813	0.2221	0.1283	-0.0235	0.3146	-0.0769	-0.0118
B glucan content	0.0707	0.1356	0.0134	0.1655	-0.0683	-0.1209	-0.0393	-0.0251	0.2123
Gluten content	-0.0664	-0.6947	-0.5178	-0.5363	-0.4518	-0.3042	-0.0294	-0.3746	0.1727
Gluten spread	-0.3245	-0.4745	-0.2438	-0.4424	-0.2712	-0.2375	-0.0037	-0.3551	0.0356
Gluten Index	0.4342	0.5465	0.3302	0.4753	0.3487	0.2916	-0.0987	0.3913	-0.1127
Zeleny sedimentation	0.4193	0.2985	0.2716	0.1965	0.3173	0.2889	-0.1312	0.3935	-0.1957
Dough Development Time	0.4750	0.3009	0.1772	0.2649	0.1780	0.1418	-0.2500	0.1435	0.1344
Dough Stability	0.4991	0.3048	0.0927	0.3285	0.1185	0.1151	-0.2383	0.2794	0.0369
Dough Softening at 12 min	-0.5288	-0.3013	-0.1300	-0.2981	-0.1549	-0.1432	0.2879	-0.1817	-0.1816
Water absorption	0.1807	-0.2287	0.0315	-0.3165	-0.0041	-0.0324	-0.1468	-0.4374	0.3456
Farinograph quality number	0.5060	0.2559	0.1341	0.2368	0.1198	0.0834	-0.2462	0.1429	0.2028

$r_{5\%} = 0.2050$, $r_{1\%} = 0.2673$, $r_{0.1\%} = 0.3375$
